# hnRNP E1 Regulates HPV16 Oncogene Expression and Inhibits Cervical Cancerization

**DOI:** 10.3389/fonc.2022.905900

**Published:** 2022-06-21

**Authors:** Li Song, Rui Mao, Ling Ding, Zhiqiang Tian, Mingxuan Zhang, Jiahao Wang, Ming Wang, Yuanjing Lyu, Chunliang Liu, Meijuan Feng, Haixia Jia, Jintao Wang

**Affiliations:** ^1^ Department of Epidemiology, School of Public Health, Shanxi Medical University, Taiyuan, China; ^2^ Questrom School of Business, Boston University, Boston, MA, United States; ^3^ Shanxi Bethune Hospital, Shanxi Academy of Medical Sciences, Tongji Shanxi Hospital, Third Hospital of Shanxi Medical University, Taiyuan, China; ^4^ Tongji Hospital, Tongji Medical College, Huazhong University of Science and Technology, Wuhan, China

**Keywords:** hnRNP E1, cervical cancerization, HPV16 E6, HPV16 E2/E6 ratio, regulation

## Abstract

hnRNP E1 (heterogeneous nuclear ribonucleoprotein E1) is an important RNA-binding protein (RBPs) that plays a vital role in tumor development. Human papillomavirus 16 (HPV16) contains numerous sites that can bind to RNA/DNA and may be modified by multiple RBPs, which contribute to HPV gene expression and HPV-associated cancer development. However, the effects of hnRNP E1 on HPV16 oncogenes in the development of cervical lesions remain unclear. A total of 816 participants with different grades of cervical lesions were enrolled in a community-based cohort established in Shanxi Province, China. The Gene Expression Omnibus (GEO) and The Cancer Genome Atlas (TCGA) databases were used to analyze the association between hnRNP E1 mRNA expression and cervical lesions. Cells with up_ and down_regulated hnRNP E1 were established. hnRNP E1 functions were evaluated using cell counting kit-8, flow cytometry analyses, and chromatin immunoprecipitation sequencing. Our results showed that hnRNP E1 expression was linearly dependent on the severity of the cervical lesions. Low expression of HPV16 E2, high expression of E6, and a low ratio of E2 to E6 could increase the risk of cervical lesions. hnRNP E1 expression was correlated with HPV16 oncogene expression. hnRNP E1-relevant genes were involved in the dopaminergic synapses, Wnt signaling pathway, gnRH secretion, and mTOR signaling pathway. hnRNP E1 significantly inhibited cell proliferation, induced apoptosis, arrested the cell cycle at the G0/G1 stage, and decreased HPV16 E6 expression. Our results indicate that hnRNP E1 could downregulate HPV16 E6 oncogene expression and inhibit cervical cancerization, which sheds new light on preventing the carcinogenicity of HPV across a range of diseases by regulating RNA-binding proteins.

## Introduction

Cervical cancer is the fourth most frequently diagnosed cancer in women globally (1). In contrast to the downward trends in developed countries, the incidence of cervical cancer in China has increased significantly. It is estimated that by 2020, there will be 0.11 million new cases and 0.06 million deaths in China (2). Shanxi Province, China has a notably high incidence of cervical cancer cases, with 5.42 associated deaths per 100,000 women in 2014 (3), which was about two times more than the national average (4).

High-risk human papillomavirus (HPV) persistent infection, especially HPV16 infection, accounts for more than 50% of HPV cases (5) and plays an important role in cervical carcinogenesis (6). The HPV16 genome comprises approximately 8000 bp of double-stranded circular DNA that can be divided into early genes (E1, E2, E4, E5, E6, and E7), late genes (L1 and L2), and long control regions. HPV16 E6, which induces and maintains cellular transformation, is an important oncogenic protein. Specifically, E6 and E7 expressions are necessary to drive proliferation in infected cells and for progression to high-grade lesions and cancer development. HPV16 E2 is considered to be the main inhibitor of E6 and E7 oncogene expression (7, 8). Integration of the HPV16 genome into host chromosomes is a vital event in cervical carcinogenesis, which usually causes disruption of E2, loss of regulation of E6, and subsequently E6 overexpression (9). Therefore, E2 and E6 play an important role in HPV integration and carcinogenesis and have attracted extensive attention as key genes for integration. However, the regulation of HPV gene expression depends on intracellular RNA processing and is usually modified by RNA binding proteins (RBPs) (10).

As RBPs, heterogeneous nuclear ribonucleoproteins (hnRNPs), contribute multiple functions to nucleic acid metabolism through post-transcriptional regulation (11). hnRNP E1 is a member of the hnRNP family and contains three K homology (KH) domains. It is required to achieve greater RNA/DNA binding affinity and specificity (12). HPV16 has two characteristic promoters, early promoter p97, and late promoter p670. In addition, the HPV16 genome covers 5′splice sites, 3′splice sites, and two polyadenylation sites. These sites provide the structural basis for specific binding to a variety of RBPs and their protein complexes (13). Studies have shown that hnRNP E1 is involved in multiple pathological processes (14, 15). hnRNP E1 expression is associated with numerous tumor types, such as liver cancer (16), pancreatic cancer (17), gastrointestinal adenocarcinomas (18), prostate cancer (14), and thyroid carcinoma (19). However, to the best of our knowledge, the association between hnRNP E1 expression and cervical cancerization remains unclear. Pillai et al. (20) used an immunohistochemical method to detect hnRNP E1 in cervical tissue with a small sample size. The results showed that the expression level of hnRNP E1 decreased gradually with the increase in the severity of the disease. Our previous study showed that high expression of hnRNP K, which is similar to hnRNP E1 in structure and function, could increase the risk of cervical lesions (21). Collier et al. (22) found that hnRNP E1 inhibited the translation of L2 mRNA of the HPV16 late gene. However, the association between hnRNP E1 and HPV16 and the progression of cervical carcinogenesis has not been reported.

Based on the specificity with which the unique KH structural domains of hnRNP E1 bind with RNA/DNA, considering that HPV16 provides RNA/DNA binding sites, we hypothesized that hnRNP E1 may be crucial to HPV16 oncogenes expression and cervical carcinogenesis. Our previous population-based results showed that hnRNP E1, HPV16 E2, and E6 are closely linked to cervical cancer development (23). In the present study, we analyzed the expression changes of hnRNP E1 in different cervical pathological stages. We further explored the potential function and mechanism of hnRNP E1 in cervical lesions *in vitro*. Our findings may serve as a foundation for elucidating novel molecular targets against HPV16 and new prognostic and predictive biomarkers for cervical cancer.

## Materials and Methods

### Study Population

**Figure 1:**
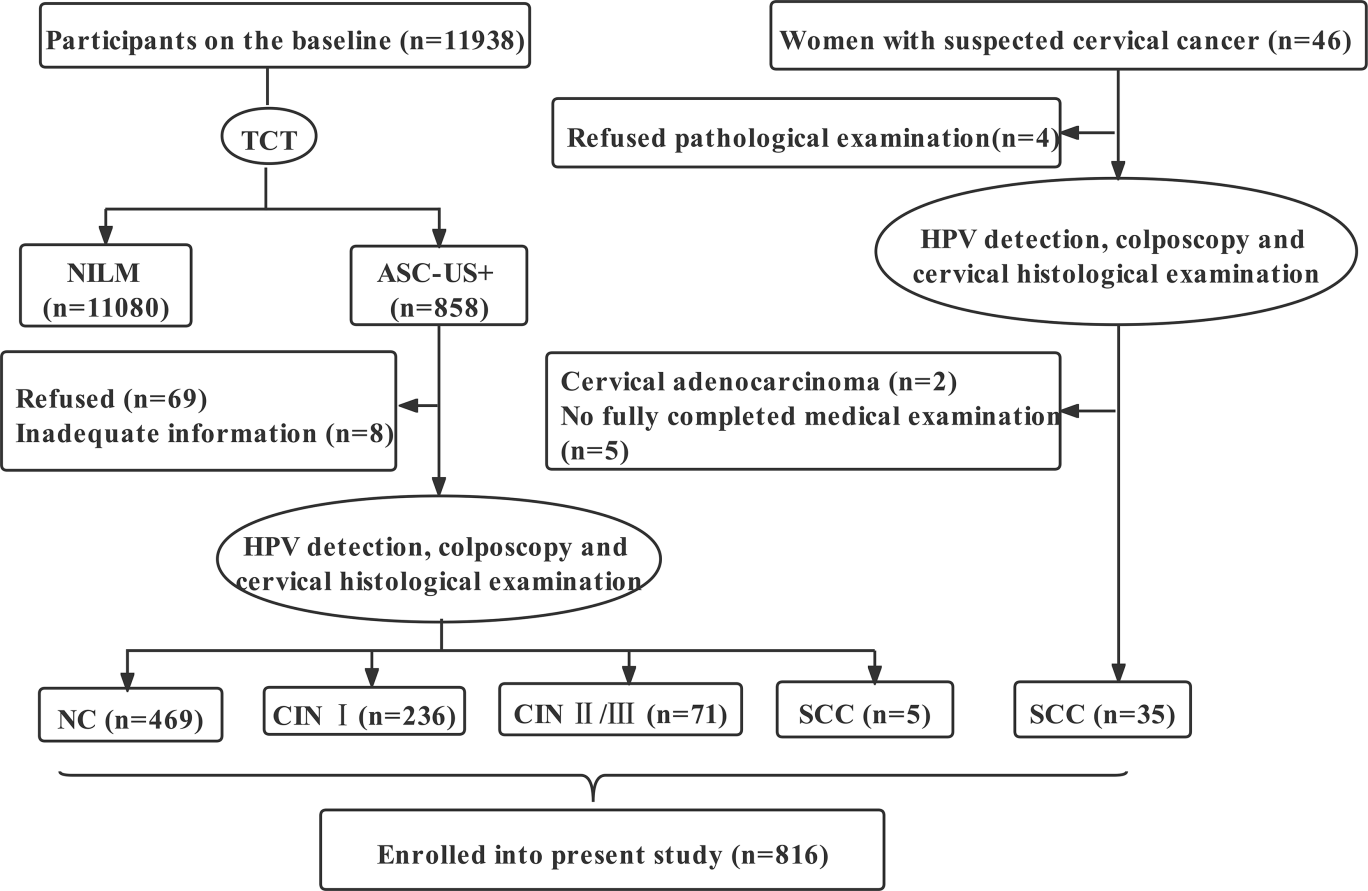
Flowchart of participants in the study. TCT, ThinPrep cytologic test; NILM, negative for intraepithelial lesion or malignancy; ASC-US+, atypical squamous cells of undetermined significance and above; NC, normal cervical; CIN I, low-grade cervical intraepithelial neoplasm; CIN II/III, high-grade cervical intraepithelial neoplasm; SCC, squamous cell carcinoma; HPV, human papillomavirus.

There were 11984 participants, including 11938 women from the community cohort established in Jiexiu City and Yangqv County, Shanxi Province, China from June to September 2014, and 46 women suspected of cervical cancer from the Shanxi Cancer Hospital. All participants followed inclusion criteria: a) married, b)18-65 years, c) had resided in Shanxi for at least 1 year, d) volunteered to participate in the study, and the exclusion criteria were: a) pregnancy, b) history of hysterectomy or cervical conization, current or prior malignancy, c) had other tumors, d) received chemotherapy or radiotherapy. After completing the ThinPrep cytologic test (TCT) of 11,938 women, 858 women were diagnosed with atypical squamous cells of undetermined significance and above (ASC-US+). Then, after 77 women were excluded, 781 women with ASC-US+ underwent HPV genotyping and histopathological examination. Of the 781 women, 469 were diagnosed by pathology as normal cervical (NC), 236 as low-grade cervical intraepithelial neoplasm (CIN I), and 71 as high-grade cervical intraepithelial neoplasm (CIN II/III) and 5 as squamous cell carcinoma of the cervix (SCC). Meantime, we collected 35 SCC patients diagnosed by pathology from the hospital. Ultimately, 816 participants were involved in the study (**Figure 1**). All participants signed informed consent, and the study was approved by the institutional review committee of Shanxi Medical University (2013–003). No potentially identifiable human images or data are presented in this study.

### Data and Sample Collection

All participants were interviewed face-to-face to collect information about sociodemographic characteristics, personal hygiene behavior, lifestyle, menstrual state, sexual life, and history of the personal disease through a structured questionnaire. Cervical swabs were collected with a cervical brush and stored at 4°C and completed HPV typing within 24 hours. Cervical biopsy specimens were collected and stored in a -80°C refrigerator immediately.

### HPV Detection

HPV16 infection assay by flow-through hybridization technology, DNA extraction, and HPV genotyping have previously been described in detail (24, 25). Briefly, HPV-DNA in cervical swabs was extracted by HPV-DNA extraction kit and amplified by PCR. HPV genotyping was performed using an HPV Geno Array Test Kit (HybriBio Ltd, Chaozhou, China) according to the manufacturer’s instructions. Twenty-one HPV genotypes can be identified. In the study, HPV16 positive was defined as the participants with HPV 16 single infection or multiple infections with other HPV genotypes.

### Cell Culture

Cervical cancer cell lines (SiHa and C33A) were obtained from the Chinese Academy of Sciences (Beijing, China). SiHa cells were cultured in high glucose Dulbecco’s modified eagle medium (Hyclone, USA), and the minimum essential medium (Boster Inc, China) was used to culture C33A cells. All the cell lines were supplemented with 10% fetal bovine serum of 5% CO_2_ at 37°C.

### Plasmid Transfection

hnRNP E1 cDNA plasmids (Genechem Co., Ltd., Shanghai, China) and hnRNP E1 shRNA (Sangon Biotech, Co., Ltd., Shanghai, China) were used to up_ and down_ regulate the expression of hnRNP E1, respectively. When the cell confluence reached 75%, the transfection complex composed of plasmid, transfection reagent (Roche Applied Science, Germany), and culture medium were added to the 6-well plates. DNA plasmids encoding Green fluorescent protein (GFP) were performed to establish the best transfection efficiencies and conditions.

### Western Blotting

Proteins were extracted from cells or tissues and added to the RIPA buffer. Cracking on ice for 30_60 minutes. The supernatant was collected by centrifugation at 4°C and 12000 rpm for 15 minutes. BCA protein assay kit (Boster Inc, China) was used for protein quantification. SDS-PAGE gel electrophoresis was performed. Then, the protein was transferred to the nitrocellulose membrane and 5% skimmed milk powder blocked the nonspecific antigen. The primary antibody was added and incubated at 4°C overnight. After washing the membrane, horseradish peroxidase-labeled IgG (secondary antibody) was added at 37 °C for 1h. Densitometric analysis was performed by Image Lab. In the assay, antibodies included rabbit anti-hnRNP E1 (Abcam, UK. ab74793, 1:1000), mouse anti-HPV16 E2 (Abcam, UK. ab17185, 1:1000), mouse anti-HPV16 E6 (Abcam, UK. Ab70, 1:1000), mouse anti-β-actin (Boster Inc, China, 1:300).

### Quantitative Real-Time RT-PCR

TRIzol Reagent (Invitrogen, CA, USA) was used for extracting total RNA from cultured cells. cDNA was prepared by reverse transcription using TransScript one-step gDNA Removal and cDNA Synthesis SuperMix kit (Transgen Biotech, China). Quantitative real-time RT-PCR (RT-qPCR) was carried out using the QuantiNova SYBR Green PCR kit (QIAGEN GmbH, Germany). PCR was carried out following the manufacturer’s protocol. The PCR primer information was shown in **Table S1**. β-actin was used as the internal control. The relative mRNA levels were defined by using the 2^-△△Ct^ method.

### Cell Proliferation, Cycle, and Apoptosis Assays

10μl CCK8 stock solution (Dojindo Laboratories, Japan) were added to each cultured 96-well plate at the stages of post-transfection 12h, 24h, 36h, 48h, and 72h, further incubated for 2h at the 37°C. The absorbance value at 450 nm was measured by a microplate reader. To analyze the cell cycle, cultured cells were collected, counted, and added 500µl 70% pre-cooling ethanol to centrifugated cells, overnight at 4°C. The next day, centrifugated cells were treated with 500μl RNaseA/Propidium Iodide and kept away from light at 25°C for 30 min. Subsequently, the cell cycle was detected by flow cytometry (FCM). Cell apoptosis was detected using an Annexin V-APC/Propidium Iodide apoptosis detection kit (KGA1030, KeyGEN Biotech, China), then, cell proliferation indexes (PI) were evaluated by a formula of (S+G2/M)÷(G0/1+S+G2/M)×100%.

### Chromatin Immunoprecipitation Sequencing and Bioinformatics Analysis

Chromatin immunoprecipitation (ChIP) experiments were performed on SiHa cells that had not been treated with any plasmid. The SimpleChIP Enzymatic Chromatin IP Kit (no. 9003; Cell Signaling Technology, Danvers, MA, USA) was used to prepare cross-linked chromatin for ChIP. Briefly, the cells were fixed with 1% formaldehyde for 10 min, followed by cell and nuclear lysis. Cross-linked DNA was sonicated and ranged in size from 200 to 900 bp. DNA/protein complexes were immunoprecipitated overnight using anti-hnRNP E1 (#8534, Cell Signaling Technology, Danvers, MA, USA) antibodies. After reverse cross-linking and DNA purification, enriched DNA was detected by PCR. The primers used were listed in **Table S1**. The quality control analysis of the ChIP experiment was presented in **Figure S1**. Sequencing was performed at BGI-Shenzhen (Shenzhen, China) using an BGISeq-500 system. The authors acknowledge that the data presented in this study must be deposited and made publicly available in an acceptable repository, prior to publication. Frontiers cannot accept a manuscript that does not adhere to our open data policies.

The raw data were aligned to hg19 using SOAPaligner/SOAP2 (26). Peak calling was conducted by MACS (Model-based Analysis of ChIP-Seq) (27). The Gene Ontology (GO) terms (28) and Kyoto Encyclopedia of Genes and Genomes (KEGG) pathway analyses (29) were performed in R software using the “clusterProfiler” to show enrichment closely related to hnRNP E1_relevant genes. A two‐sided Fisher’s exact test and the “enrichplot” and “ggplot2” packages were used to visualize the top 10 enriched terms and KEGG pathways.

### Statistical Analysis

Analyses were performed using SPSS 22.0, R 4.0 and graphics were generated using GraphPad Prism 7.0. Significance testing between groups was performed by analysis of variance (ANOVA), Bonferroni’s multiple comparison tests, The *post hoc* Least Significant Difference (LSD) test, the Kruskal-Wallis H test, and the Chi-square test. Possible associations were analyzed using Spearman’s rank-order correlation. Kaplan-Meier survival analyses were performed in R 4.0 software using the “survival” and “survminer” to assess the relevance of hnRNP E1 expression and prognosis of the patient with cervical cancer. All analyses were two-sided and α=0.05.

The raw gene expression profiles of GSE9750 and GSE75132 were downloaded from the Gene Expression Omnibus (GEO) and used to analyze the expression of hnRNP E1 in cervical cancer and its relationship with HPV. GSE9750, containing 24 normal cervical samples and 33 cervical cancer samples. GSE75132 included data from six participants with persistent HPV16 infection and participants without HPV. The mRNA expression and clinical information of 304 patients with cervical cancer were downloaded from The Cancer Genome Atlas (TCGA) portal (https://portal.gdc.cancer.gov/).

## Results

### 1. Demographic Characteristics of Participants and Factors Related to Cervical Lesions

We analyzed the demographic characteristics and factors related to cervical lesions of 816 participants. The average age of 816 women was 47 ± 12 years (range 19_65 years). Of all participants, 79.5% had received education in junior high school and above. The distribution of marital status, age, education level, and occupation among different cervical lesions groups was not significant (*P*>0.05); however, early age of first sexual intercourse, multiple gravidities, low bathing frequency, and low vaginal cleaning frequency were found to be associated with increased risk of CIN and cervical cancer(*P*<0.05).

### 2. HPV16 Gene Expression in Multistage Cervical Cancerization

The prevalence of HPV16 in NC, CIN I, CIN II/III, and SCC were 8.53%, 14.41%, 40.85%, and 67.50%, respectively, with significant differences, and showed an upward trend with the aggravation of cervical lesions (*χ^2^_trend_* =113.560, *P*<0.001). The results were displayed in **Table 1**. Subsequent results showed that HPV16 E2 protein levels in the NC and CIN I groups were significantly higher than those in the CIN II/III and SCC groups. The HPV16 E6 protein levels in the NC and CIN I groups was significantly lower than those in the CIN II/III and SCC groups (**Table 1**). There were significant differences in the ratio of E2 to E6 in different groups (*H*=71.392, *P*<0.001), showing a decreasing trend from NC to CIN I, CIN II/III, and SCC (**Table 1**).

**Table 1:** Associations between HPV16 genes expression and cervical lesions.

Group	N	HPV16 infectionn (%)	HPV16 E2 M(Q)*	HPV16 E6 M(Q)*	Ratio of E2/E6 M(Q)*
NC	469	40(8.53)	1.72(0.28)^a^	0.18(0.08)^a^	9.89(4.75)^a^
CIN I	236	34(14.41)	1.71(0.34)^a^	0.17(0.06)^ab^	9.39(3.20)^a^
CIN II/III	71	29(40.85)	1.08(0.86)^bc^	0.47(0.78)^c^	2.13(3.72)^bc^
SCC	40	27(67.50)	0.86(0.48)^c^	1.25(0.67)^d^	0.69(0.85)^b^
		*χ^2^_trend_=*113.56*0*, *P*<0.001	*H^#^=*46.207*, P < 0.001*	*H* ^#^=66.848, *P* < 0.001	*H* ^#^=71.392, *P* < 0.001

*median (quartile range); a/b/c/d, different letters indicate significant differences at least P<α’ (α’=0.05/6 = 0.0083); ^#^overall comparison among different groups.

### 3. hnRNP E1 Expression in Multistage Cervical Cancerization and Associations With HPV16 E2 and E6

**Figure 2:**
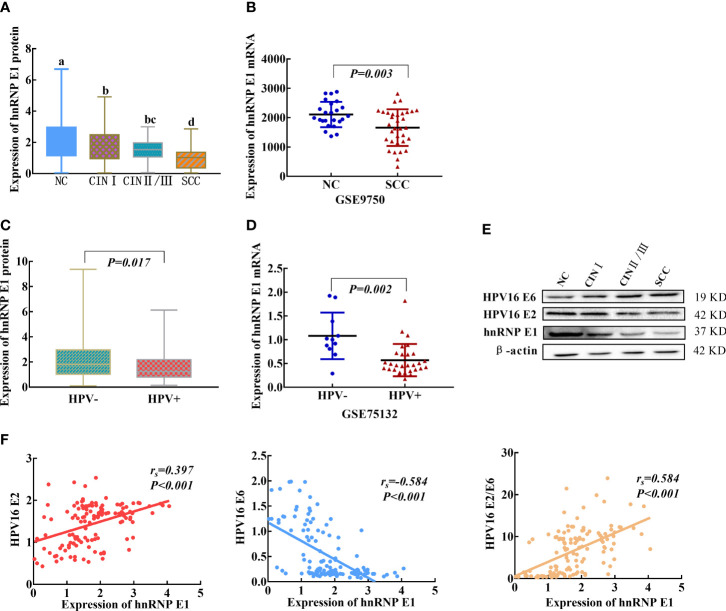
hnRNP E1 expression and correlations with HPV16 E2 and E6 in cervical lesions. **(A)** hnRNP E1 protein expression levels in the clinical samples. **(B)** The hnRNP E1 mRNA expression levels in NC and SCC samples, data were obtained from GEO dataset. **(C)** The hnRNP E1 protein expression levels in HPV+ and HPV- samples. **(D)** The hnRNP E1 mRNA expression levels in HPV+ and HPV- samples, data were available from GEO dataset. **(E)** hnRNP E1, HPV16 E2 and E6 expression levels was detected using western blotting in cervix tissues. β-actin was used as loading control. **(F)** Correlations between hnRNP E1, HPV16 E2 and E6 in cervical lesions. a/b/c/d, different letters indicate significant differences at least *P*<α’(α’=0.05/6 = 0.0083). HPV+ represents HPV-positive samples, and HPV- represents HPV-negative samples.

To explore hnRNP E1 expression patterns in cervical lesions, hnRNP E1 protein expression was detected using western blotting. With the progress of cervical lesions, hnRNP E1 protein expression levels gradually decreased (**Figure 2A**). The average hnRNP E1 expression level in NC (2.081 ± 1.708, n=469) was 2.30 times higher than that in SCC (0.906 ± 0.844, n=40). Moreover, data from the GEO dataset (GSE9750) showed that hnRNP E1 mRNA was more highly expressed in NC samples than in SCC samples (**Figure 2B**). We further investigated the associations between hnRNP E1 and HPV in cervical lesions and found that the expression levels of hnRNP E1 protein or mRNA in the HPV-negative group were significantly higher than those in the HPV-positive group (*P*<0.05), as shown in **Figures 2C**, **D**. Spearman’s rank correlation analysis showed positive correlations between hnRNP E1 expression and HPV16 E2 (*r_s_* =0.397, *P*<0.001) and the ratio of HPV16 E2 to E6 (*r_s_* =0.584, *P*<0.001) and a negative correlation between hnRNP E1 and HPV16 E6 (*r_s_* =-0.584, *P*<0.001), as shown in **Figures 2E, F**.

### 4. Correlations Between hnRNP E1 and Prognosis of the Patient With Cervical Cancer Based on TCGA Database

**Figure 3:**
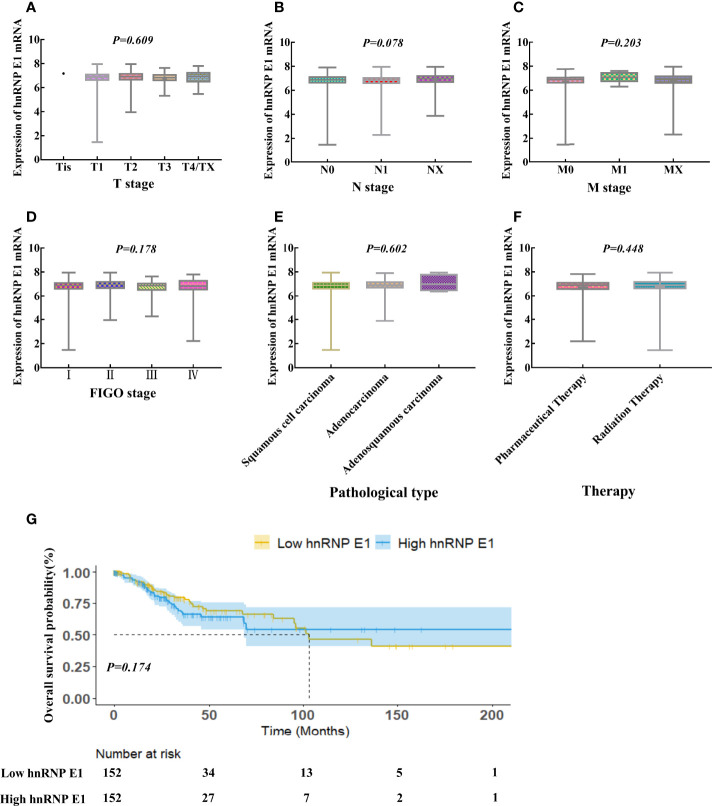
Correlations between hnRNP E1 and prognosis of the patient with cervical cancer based on TCGA database. hnRNP E1 mRNA expression levels in the different T stage **(A)**, N stage **(B)**, M stage **(C)**, International Federation of Gynecology and obstetrics (FIGO) stage **(D)**, Pathological type **(E)** and Therapy **(F)**. **(G)** Kaplan-Meier survival curves were performed for the overall survival probability.

We further analyzed the relationship between hnRNP E1 expression and clinicopathological characteristics in cervical cancer based on the TCGA database. The Kruskal-Wallis rank-sum test showed that there was no significant correlation between hnRNP E1 expression and T stage (*P=0.609*), N stage (*P=0.078*), M stage (*P=0.203*), International Federation of Gynecology and Obstetrics (*FIGO*) stage (*P=0.178*), pathological type (*P=0.602*) and therapy (*P=0.448*) of cervical cancer (**Figures 3A–F**). To better understand the relevance of hnRNP E1 expression and the prognosis of the patient with cervical cancer, we performed Kaplan-Meier survival analysis. Patients were divided into high-hnRNP E1 and low-hnRNP E1 groups based on the median value. Results showed that the overall survival rate was not significant between the high-hnRNP E1 and low-hnRNP E1 groups *(P>0.0*5, **Figure 3G**). Although our results have revealed the subtle prognostic value of hnRNP E1, compared with the low expression of hnRNP E1, high expression of hnRNP E1 has a better prognosis.

### 5. Functional Enrichment Analysis of Binding Sites Relative to hnRNP E1

**Figure 4:**
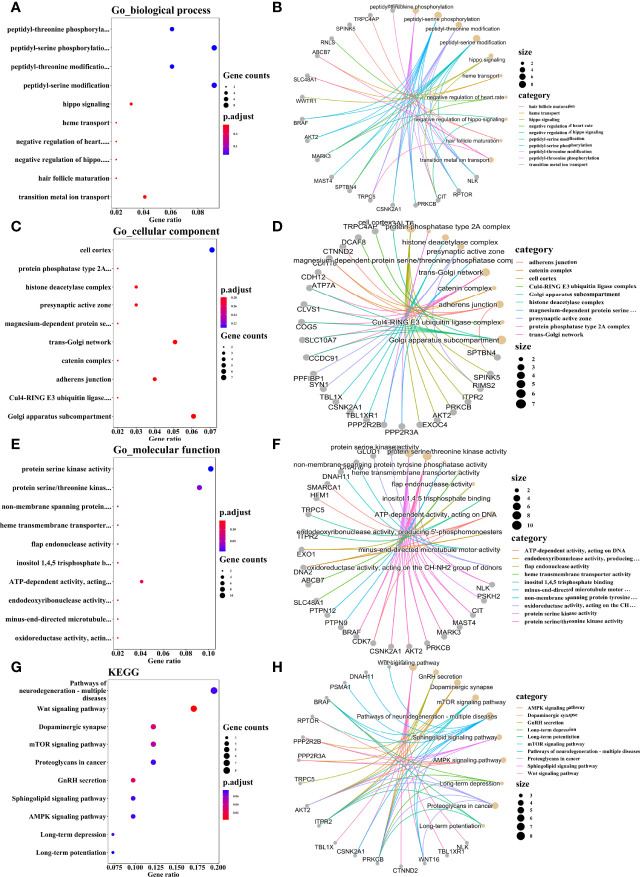
GO and KEGG pathway enrichment analyses of hnRNP E1 related genes. Note: BP, biological process. CC, cellular components. MF, molecular function. GO, Gene Ontology. KEGG, Kyoto Encyclopedia of Genes and Genomes.The top 10 significant BP **(A)**, CC **(C)**, MF **(E)**, and KEGG pathways **(G)**. Construction of the enrichment BP_genes network **(B)**, CC_genes network **(D)**, MF_genes network **(F)**, and pathways_genes network **(H)**. **(A, C, E, G)** The x axis reflects gene ratio, and the y axis represents the KEGG and GO terms. The node size represents the gene count. The node colors represent adjusted *P* values. **(B, D, F, H)** The size of the circle indicates the number of enriched genes. Lines represent linkages of genes and enriched terms.

Based on the main purpose of this study was to explore the role of hnRNP E1 on the regulation of HPV16 oncogene expression in cervical cancerization, we conducted ChIP-seq in the untreated SiHa cell line. A total of 121 potential targets for hnRNP E1 across the human genome in SiHa cells were identified, including 357 binding sites (peaks). The average peak length was 161 bp, and the length was mainly distributed between 100_500 bp. To further analyze the potential biological functions of annotated genes related to hnRNP E1, GO and KEGG enrichment analyses were performed, and the screening criteria was *P-value*<0.3. According to the functional annotation in the GO database, the most significant biological process (BP) terms were peptidyl-threonine phosphorylation, peptidyl-serine phosphorylation, peptidyl-threonine modification, and peptidyl-serine modification, and Hippo signaling. When focusing on cellular components (CC), the most highly represented categories were the cell cortex, protein phosphatase type 2A complex, histone deacetylase complex, presynaptic active zone, and magnesium-dependent protein serine/threonine phosphatase complex. The main functional categories of molecular function (MF) were related to protein serine kinase activity, on-membrane spanning protein tyrosine phosphatase activity, heme transmembrane transporter activity, flap endonuclease activity, and inositol 1,4,5 trisphosphate binding. GO enrichment terms of BP, CC, and MF for hnRNP E1 annotated genes are shown in **Figures 4A, C, E**. Based on the KEGG pathway enrichment analysis, hnRNP E1_relevant genes were involved in the dopaminergic synapse, Wnt signaling pathway, gnRH secretion, mTOR signaling pathway, pathways of neurodegenerative diseases, sphingolipid signaling pathway, AMPK signaling pathway, proteoglycans in cancer, and long-term depression. (**Figure 4G**). The top 10 most enriched functions were obtained to construct a network (**Figures 4B, D, F, H**). It was worth noting that we found that hnRNP E1 relevant genes were enriched in HPV infection pathway. These results suggested that hnRNP E1 may play a key role in HPV-induced cervical lesions. Detailed information was listed in **Table S2**.

### 6. hnRNP E1 Inhibits Cervical Cancer Cell Proliferation *in Vitro*

**Figure 5:**
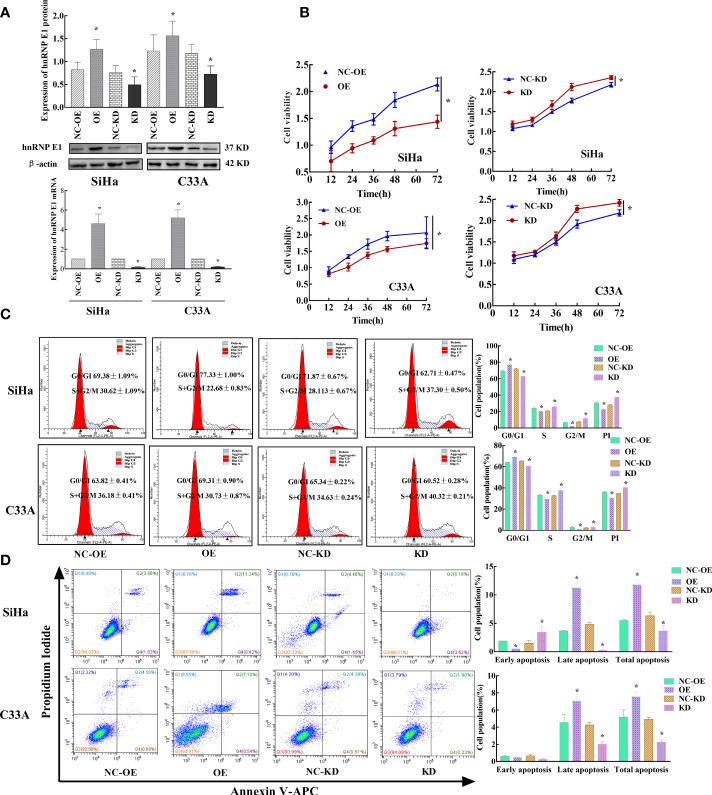
Effects of hnRNP E1 on cell biological function in cervical cancer cell lines. Note: OE indicates hnRNP E1 overexpression group and NC-OE indicates the relative control group. KD indicates hnRNP E1 knockdown group and NC-KD indicates the relative control group. *P < 0.05. **(A)** Stably transfected hnRNP E1-modified cervical cancer cells were identified by RT-qPCR and western blotting. **(B)** hnRNP E1 overexpression strongly suppressed cell viabilities in SiHa and C33A cells. hnRNP E1 knockdown markedly enhanced cell viabilities in SiHa and C33A cells. **(C)** hnRNP E1 arrests the cell cycle from the G0/G1 to the S phase. **(D)** hnRNP E1 promotes cell apoptosis.

To further explore the biological role of hnRNP E1 in cervical cancer, cultured SiHa, and C33A cell lines were transduced with hnRNP E1 overexpression plasmid or shRNA. OE indicates the hnRNP E1 overexpression group and NC-OE indicates the relative control group. KD indicates the hnRNP E1 knockdown group and NC-KD indicates the relative control group. Overexpression and knockdown efficiencies of hnRNP E1 were confirmed by RT-qPCR and western blotting (**Figure 5A**). As shown in **Figure 5B**, compared with the control group, overexpression of hnRNP E1 (OE) in SiHa and C33A cells resulted in decreased cell viability (NC-OE, *P*<0.05), and it was found that the inhibition rate of viability in hnRNP E1 overexpression was significantly higher than that of the control cells after transfection (0.36% ± 0.02% vs. 0.05% ± 0.04%, *P*<0.05; 0.28% ± 0.03% vs. 0.10% ± 0.03%, *P*<0.05). Conversely, knockdown of hnRNP E1 in SiHa and C33A cells significantly promoted cell growth with an increase in the cell proliferation rate at 48 h after transfection (0.23% ± 0.01% vs. 0.03% ± 0.03%, *P*<0.05; 0.22% ± 0.02% vs.0.03% ± 0.02%, *P*<0.05).

Next, the cell cycles were analyzed by FCM. As shown in **Figure 5C**, overexpression of hnRNP E1 increased the percentage of G0/G1 phase cells in SiHa and C33A cells (77.33% ± 1.00% vs. 69.38% ± 1.09%, *P*<0.05; 69.31% ± 0.90% vs.63.82% ± 0.41%, *P*<0.05) and reduced the proportion of S/G2/M cells and PI (22.68% ± 0.83% vs.30.62% ± 1.09%, *P*<0.05; 30.73% ± 0.87% vs.36.18% ± 0.41%, *P*<0.05). Conversely, hnRNP E1 knockdown in SiHa and C33A cells markedly attenuated the percentage of G0/G1 cells (62.71% ± 0.47% vs. 71.87% ± 0.67%, *P*<0.05; 60.52% ± 0.28% vs. 65.34% ± 0.22%, *P*<0.05), but increased the proportion of S/G2/M phase and PI (37.20% ± 0.50% vs. 28.13% ± 0.67%, *P*<0.05; 40.32% ± 0.21% vs. 34.63% ± 0.24%, *P*<0.05). These results suggest that hnRNP E1 affects cell cycle progression and G0/G1 phase arrest. Additionally, we compared the changes in cell cycles and proliferation index of SiHa and C33A cells modified by hnRNP E1 and found that the changes in PI in SiHa cells were higher than those in C33A cells (**Figure S2A**). To understand hnRNP E1-induced cell apoptosis, we performed FCM on hnRNP E1-intervened SiHa and C33A cell lines. These results indicated that the total apoptotic rate was significantly increased after the upregulation of hnRNP E1 in SiHa and C33A cell lines (11.81% ± 0.77% vs. 5.52% ± 0.11%, *P*<0.05; 7.57% ± 0.53%, vs. 5.16% ± 0.88%, *P*<0.05), as shown in **Figure 5D**. Conversely, knockdown of hnRNP E1 in SiHa and C33A cells considerably reduced the total apoptotic rate (3.64% ± 0.95% vs. 6.33% ± 0.67%, *P*<0.05; 2.25% ± 0.27% vs.4.92% ± 0.25%, *P*<0.05). These results were consistent with our work in this population and indicated that hnRNP E1 played a tumor suppressor role in cervical cancer. Furthermore, we analyzed changes in the apoptotic rate of SiHa and C33A cells modified by hnRNP E1 and found that the changes in the early and late apoptotic rate of SiHa cells were superior to those of C33A (**Figure S2B**).

### 7. hnRNP E1 Effects on HPV16 Oncogene and Subsequent Changes in Cell Biological Function

**Figure 6:**
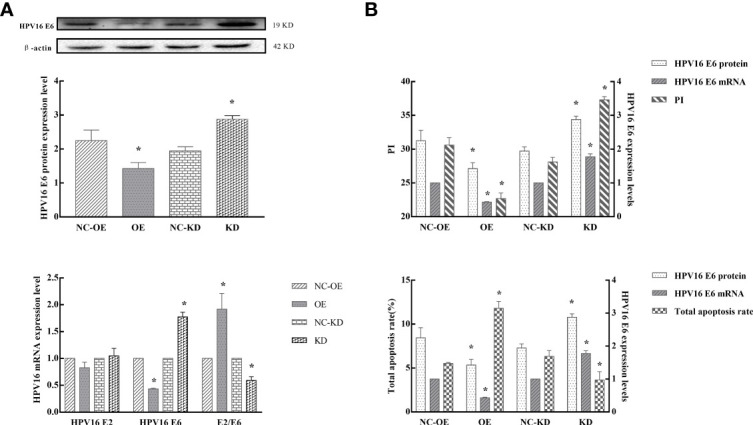
hnRNP E1 effects on HPV16 oncogene and subsequent changes in cell biological function. Note: OE indicates hnRNP E1 overexpression group and NC-OE indicates the relative control group. KD indicates hnRNP E1 knockdown group and NC-KD indicates the relative control group. *P < 0.05. **(A)** The expression of HPV16 E2, and E6 were detected in SiHa cells by RT-qPCR and western blotting. **(B)** The relationship between HPV16 E6 expression, cell proliferation, and apoptosis in hnRNP E1-modified cells. The expression of HPV16 E2, and E6 were detected in SiHa cells by RT-qPCR and western blotting. The PI and total apoptosis rate were evaluated using flow cytometry analyses. As HPV16 E6 mRNA and protein expression levels decreased, the proliferation indices of SiHa cells decreased, while the total apoptosis rate increased. As HPV16 E6 expression increased, the proliferation indices of SiHa cells increased, while the total apoptosis rate decreased.

Our population-based study identified a negative correlation between hnRNP E1 and HPV16 E6 and a positive correlation between hnRNP E1 and HPV16 E2 protein expression. We confirmed that hnRNP E1 induced different biological changes in SiHa and C33A cells *in vitro*. Next, to test whether hnRNP E1 regulated HPV16 oncogene expression, HPV16 E2 and HPV16 E6 were assessed in SiHa cells. As shown in **Figure 6A**, hnRNP E1 overexpression downregulated HPV16 E6 expression at both the mRNA and protein levels but increased the ratio of HPV16 E2 to E6 at the mRNA level. Conversely, hnRNP E1 knockdown markedly enhanced HPV16 E6 expression at both the mRNA and protein levels but decreased the HPV16 E2 to E6 ratio at the mRNA level (**Figure 6A**).

We further analyzed the relationship between HPV16 E6 expression, cell proliferation, and apoptosis. The expression of HPV16 E2 and E6 were detected in SiHa cells by RT-qPCR and western blotting. The PI and total apoptosis rate were evaluated using FCM. Our results indicated that as HPV16 E6 expression decreased, the proliferation indices of SiHa cells were reduced (*P*<0.05), while the total apoptosis rate increased (*P*<0.05). Conversely, as HPV16 E6 expression increased, the PI of SiHa cells increased (*P*<0.05), while the total apoptosis rate was attenuated (*P*<0.05), especially in hnRNP E1-modified cells (**Figure 6B**). Taken together, these findings indicated that hnRNP E1 may act as a tumor suppressor by downregulating HPV16 E6 expression.

## Discussion

In this study, we reviewed and compared hnRNP E1 expression in participants with and without HPV and explored the relationship between hnRNP E1 expression levels and cervical lesion development. Mechanistically, hnRNP E1 expression may be a significant factor affecting HPV carcinogenicity by downregulating HPV16 oncogene expression and mitigating cancerization.

In the past decade, the contribution of HPV16 persistent infection to invasive cervical cancer has been well-documented (30). The HPV16 prevalence was 3.7% in women residing in Shanxi Province, which exceeded the national average (0.78%) in 2014 (31). We observed that HPV16 infection rates were 8.53%, 14.41%, 40.85%, and 67.50% in the NC, CIN I, CIN II/III, and SCC groups, respectively. This further corroborated that HPV16 infection is a crucial etiological factor for cervical lesions, especially CIN II/III and SCC. Therefore, it is essential to carry out HPV screening to prevent the occurrence and progression of cervical cancer. HPV16 E2 is crucial for transcriptional regulation, DNA replication, viral genome tethering, and viral DNA packaging (32). Xue et al. (33) found that E2 was highly expressed in CINI compared with that in CIN II/III through immunohistochemistry. The present study revealed that HPV16 E2 low expression was closely related to the development of cervical lesions, especially CIN II/III and SCC. HPV16 E6 is a multifunctional oncoprotein that mediates various biological events, such as promoting the degradation of p53, regulating the transcription of cell cycle-associated genes, activating telomerase, and contributing to immune response and cell communication (34, 35). Wang et al. (36) suggested that HPV16 E6 could promote the migration and invasion of cervical cancer cells. Our previous study combined with this study indicated that HPV16 E6 increases the risk of cervical cancerization (37). BPV E2 expression results in specific inhibition of HPV E6 gene expression in cells and considerable growth inhibition^8^. HPV16 DNA integration and virus E2 gene damage often occur in cervical carcinogenesis, suggesting that inactivation of the HPV E2 gene allows high expression of the E6 gene, which can promote the growth of cervical epithelial cells (38). Our study showed that the ratios of HPV16 E2 to E6 in CIN II/III and SCC groups were significantly lower than those in the NC and CIN I groups. This observation was supported by Choi et al. (39) who discovered that the mean HPV16 E2/E6 ratio decreased significantly following a linear trend from CIN II/III to SCC. The ratio of E2 to E6 may be a sign of cancer progression.

hnRNP E1 was downregulated in numerous tumors (40). Zhang et al. (14) reported that hnRNP E1 functions as a tumor suppressor in gastric cancer. The expression of hnRNP E1 and miRNA-3978 in peritoneal metastasis of gastric cancer was inhibited (15). Pillai et al. (20) discovered that hnRNP E1 expression decreased from 86% in CIN I to 68% in CIN II/III and 40% in cervical cancer. Pathak et al. (41) also found that hnRNP E1 expression decreased progressively from the normal cervix (100%) to squamous intraepithelial lesions (75%) and cervical cancer (52.6%). The present study showed that with the development of cervical lesions, hnRNP E1 expression decreased from NC to CIN and SCC. Although the survival analysis based on the TCGA database revealed a subtle prognosis value of hnRNP E1. These results indicated that low hnRNP E1 expression contributed to the risk of cervical lesions and promoted the progression of cervical cancerization. hnRNP E1 may be considered a biomarker for the early detection of cervical carcinogenesis.

To understand the binding capacity of hnRNP E1 at the genome-wide level, we conducted ChIP-seq in the untreated SiHa cell line. Our data further supported that hnRNP E1 relevant genes were associated with a series of GO terms related to cell proliferation, metabolism, and apoptosis. KEGG pathway enrichment analysis revealed that hnRNP E1 relevant genes were mainly involved in the Wnt signaling pathway, GnRH secretion, dopaminergic synapse, mTOR signaling pathway, pathways of neurodegeneration-multiple diseases, sphingolipid signaling pathway, and AMPK signaling pathway. The aberrant Wnt/β-catenin, mTOR, and MAPK signaling pathways facilitate cancer cell proliferation and differentiation (42–44). GnRH is known primarily as a neuroendocrine decapeptide that is essential for maintaining the reproductive state (45). Most notably, we found that hnRNP E1 relevant genes were enriched in HPV infection pathway, although the effect was not significant. These results suggested that hnRNP E1 may play a key role in HPV-induced cervical lesions.

To better understand the biological mechanism of hnRNP E1 in cervical lesions, we performed *in vitro* plasmid transfection. Our results demonstrated that hnRNP E1 inhibited cervical cancer cell proliferation, promoted apoptosis, and arrested the cell cycle in the G0/G1 phase. *In vitro* research has shown that the deletion of hnRNP E1 reduced the expression of p27, a key regulator of the cell cycle, and promoted carcinogenesis (46). Overexpression of hnRNP E1 reduced the expression of p53 (47) and played a vital role in the cell’s biological function and DNA damage response (48). In addition, we confirmed that hnRNP E1 induced different biological changes in SiHa and C33A cells *in vitro*. The changes in biological function in SiHa cells were greater than those in C33A cells. Given the key tumor-suppressive role of hnRNP E1 in cervical cancerization, it may have great therapeutic potential for cervical cancer. hnRNP E1 can inhibit the translation of HPV16 L2 mRNA *in vitro* (22). However, it remains unclear whether hnRNP E1 can regulate the HPV16 early gene. Our results demonstrated that hnRNP E1 was positively correlated with HPV16 E2 or E2/E6 ratio and negatively correlated with HPV16 E6. In addition, *in vitro* experiments verified that hnRNP E1 overexpression diminished the expression of HPV16 E6 but improved the ratio of HPV16 E2 to E6. In contrast, hnRNP E1 knockdown significantly increased HPV16 E6 expression but attenuated the ratio of HPV16 E2 to E6. The relationship between hnRNP E1 and HPV16 E2 protein in SiHa cells was not found in this study, which may be related to the fact that during HPV integration, E2 is destroyed in SiHa and may not produce functional E2 (49). E6 is one of the earliest-expressed genes following HPV infection, containing the selective binding site for hnRNP E1 (50). The KH domain in hnRNP E1 may reduce the expression of HPV16 E6 by binding with its regulatory element. Interestingly, our results showed that with a decrease in HPV16 E6 expression, the proliferation index of SiHa cells decreased, and the total apoptosis rate increased. Conversely, with the increase in HPV16 E6 expression, the proliferation indices of SiHa cells increased while the total apoptosis rate decreased, and this effect was more significant when hnRNP E1 was modified. Our findings suggest that HPV16 E6 low expression inhibits the proliferation and promotes cervical cancer cell apoptosis, and this effect may be regulated by hnRNP E1.

Our study does, however, have several limitations. First, our study was based on a cross-sectional study, prospective cohort studies can be conducted in the future to further explore the effect of hnRNP E1 on the prognosis of cervical lesions and HPV16 gene expression. Second, we only selected two cervical cancer cell lines for *in vitro* experiments, the conclusions will be further demonstrated in a variety of cervical cancer cell lines. Lastly, the *in vivo* effect of hnRNPE1 overexpression in the xenograft of cervical cancer cell lines was unclear, future relevant animal experiments will need to be done.

In conclusion, our study indicated that abnormally low expression of hnRNP E1 could promote cervical lesion development and was closely linked with HPV16 E2, E6 expression, and the E2 to E6 ratio. hnRNP E1 suppresses tumor growth *in vitro* by inhibiting proliferation and promoting apoptosis. hnRNP E1 relevant genes were significantly enriched in cervical cancer-related pathways. Moreover, hnRNP E1 significantly downregulated the expression of HPV16 E6. Upregulating hnRNP E1, particularly incorporating the control patterns of HPV16 infection, may offer advances in the control of cervical cancer. Owing to the complexity of HPV gene expression and post-transcriptional regulation, in-depth studies are needed to understand the underlying mechanisms of hnRNP E1.

## Data Availability Statement

The datasets presented in this study can be found in online repositories. The names of the repository/repositories and accession number(s) can be found below: GEO with accession GSE203023: (https://www.ncbi.nlm.nih.gov/geo/query/acc.cgi?acc=GSE203023).

## Ethics Statement

The studies involving human participants were reviewed and approved by the institutional review committee of Shanxi Medical University (2013–003). The patients/participants provided their written informed consent to participate in this study.

## Author Contributions

JTW conceived the project. RM, LD, and ZT designed the experiments. MZ and JHW performed the experiments. MW and YL contributed reagents, materials, and analysis tools. CL, MF, and HJ integrated, analyzed, and interpreted all data. JTW contributed to the supervision of the work. LS wrote the manuscript with the assistance and final approval of all authors.

## Funding

This subject is supported by the National Natural Science Foundation of China (No. 81872705, No. 81473060), the National Health and Family Planning Commission of the People’s Republic of China (No. 201402010), and the Fundamental Research Program of Shanxi Province (No.202103021224354).

## Conflict of Interest

The authors declare that the research was conducted in the absence of any commercial or financial relationships that could be construed as a potential conflict of interest.

## Publisher’s Note

All claims expressed in this article are solely those of the authors and do not necessarily represent those of their affiliated organizations, or those of the publisher, the editors and the reviewers. Any product that may be evaluated in this article, or claim that may be made by its manufacturer, is not guaranteed or endorsed by the publisher.
