# Supplementary material for: hnRNP E1 Regulates HPV16 Oncogene Expression and Inhibits Cervical Cancerization
Source: Front Oncol. 2022 Jun 21;12:905900. doi: 10.3389/fonc.2022.905900 (PMC9253288; doi:10.3389/fonc.2022.905900)
Supplement: Supplementary file 1 [file Presentation_1.pdf]

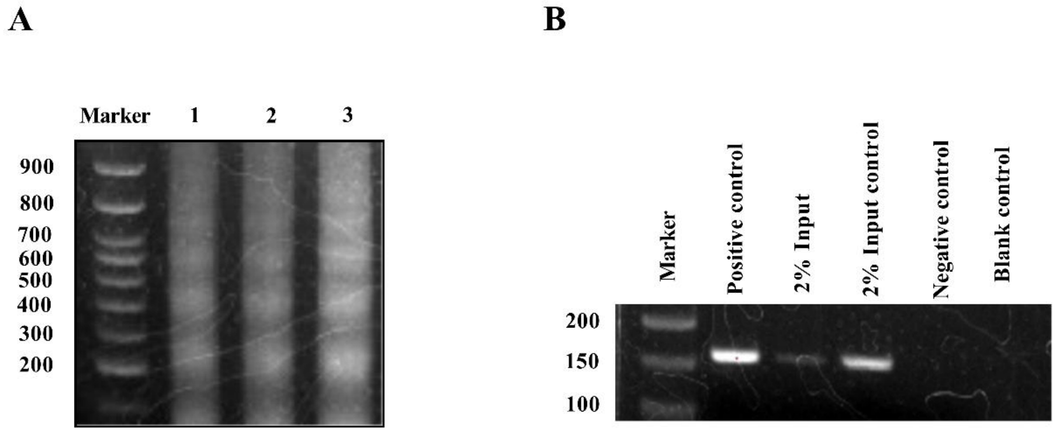

Fig. S1 Quality control analysis of ChIP experiment. (A) Distribution of purified chromosomal DNA fragments on a 1% agarose gel after sonication. Lane 1, 2, and 3 represented three repetitions. (B) Analysis of ChIP enrichment efficiency by PCR.
