# Supplementary material for: hnRNP E1 Regulates HPV16 Oncogene Expression and Inhibits Cervical Cancerization
Source: Front Oncol. 2022 Jun 21;12:905900. doi: 10.3389/fonc.2022.905900 (PMC9253288; doi:10.3389/fonc.2022.905900)
Supplement: Supplementary file 2 [file Presentation_2.pdf]

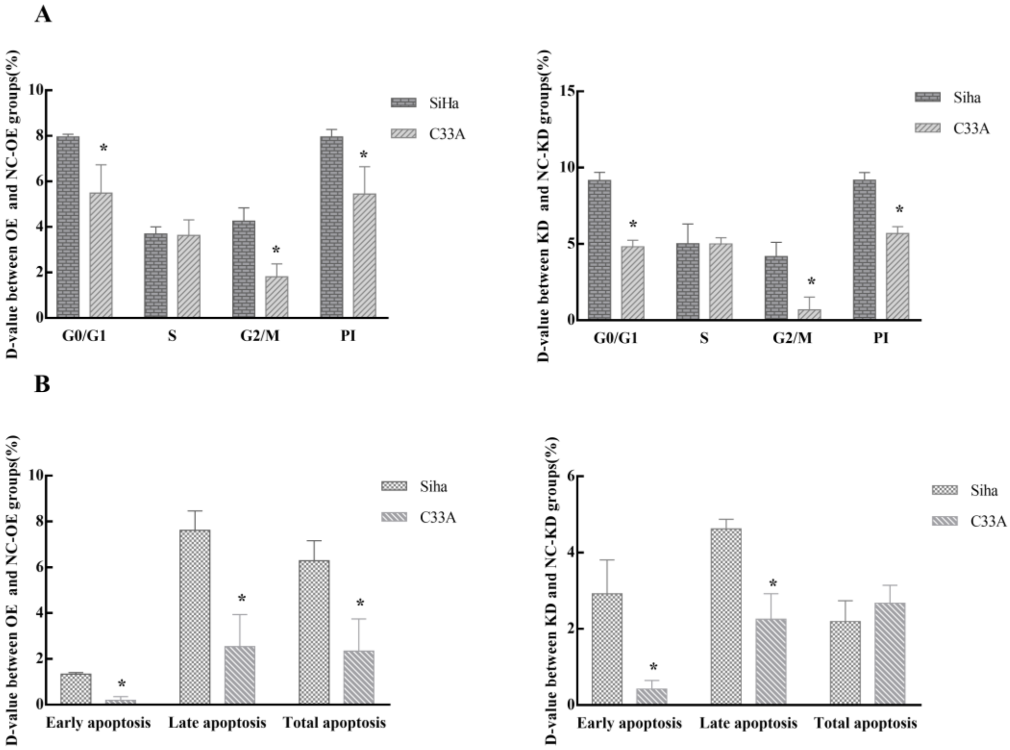

Fig. S2 Comparation of cell cycle and cell apoptotic rate changes between SiHa and C33A cells modified by hnRNP E1. (A) Comparation of cell cycle changes between SiHa and C33A cells modified by hnRNP E1. (B) Comparation of cell apoptotic rate changes between SiHa and C33A cells modified by hnRNP E1. *, P<0.05.
