# Supplementary material for: hnRNP E1 Regulates HPV16 Oncogene Expression and Inhibits Cervical Cancerization
Source: Front Oncol. 2022 Jun 21;12:905900. doi: 10.3389/fonc.2022.905900 (PMC9253288; doi:10.3389/fonc.2022.905900)
Supplement: Supplementary file 3 [file Table_1.docx]

**Table S1 Primer list of quantitative real-time RT-PCR**

| Gene | Bidirectional primer sequence（5’~3’） | Product length (bp) |
| --- | --- | --- |
| hnRNP E1 | F: TCAACAGCTCCATGACCAAC | 113 |
|  | R: GATCTTACACCCGCCTTTCC |  |
| HPV16 E2 | F: TGGAAACACATGCGCCTAGAA | 98 |
|  | R: GATACAGCCAGTGTTGGCAC |  |
| HPV16 E6 | F: AGCGACCCAGAAAGTTACCA | 134 |
|  | R: GCATAAATCCCGAAAAGCAA |  |
| β-actin | F: TGGCACCCAGCACAATGAA | 186 |
