# Supplementary material for: hnRNP E1 Regulates HPV16 Oncogene Expression and Inhibits Cervical Cancerization
Source: Front Oncol. 2022 Jun 21;12:905900. doi: 10.3389/fonc.2022.905900 (PMC9253288; doi:10.3389/fonc.2022.905900)
Supplement: Supplementary file 4 [file Table_2.docx]

**Table S2 GO and KEGG pathway enrichment analysis of the binding site relative to hnRNP E1**

| ONTOLOGY | ID | Description | Gene Ratio | Bg Ratio | P value | P. adjust | Q value | Gene ID | Count |
| --- | --- | --- | --- | --- | --- | --- | --- | --- | --- |
| MF | GO:0106310 | protein serine kinase activity | 10/98 | 360/18368 | 2.24E-05 | 0.006134 | 0.005585 | NLK/PSKH2/CIT/MAST4/MARK3/PRKCB/AKT2/CSNK2A1/CDK7/BRAF | 10 |
| MF | GO:0004674 | protein serine/threonine kinase activity | 9/98 | 386/18368 | 0.000221 | 0.030232 | 0.027526 | NLK/CIT/MAST4/MARK3/PRKCB/AKT2/CSNK2A1/CDK7/BRAF | 9 |
| MF | GO:0004726 | non-membrane spanning protein tyrosine phosphatase activity | 2/98 | 10/18368 | 0.001233 | 0.067574 | 0.061525 | PTPN9/PTPN12 | 2 |
| MF | GO:0015232 | heme transmembrane transporter activity | 2/98 | 10/18368 | 0.001233 | 0.067574 | 0.061525 | SLC48A1/ABCB7 | 2 |
| MF | GO:0048256 | flap endonuclease activity | 2/98 | 10/18368 | 0.001233 | 0.067574 | 0.061525 | DNA2/EXO1 | 2 |
| MF | GO:0070679 | inositol 1,4,5 trisphosphate binding | 2/98 | 13/18368 | 0.002115 | 0.096594 | 0.087947 | ITPR2/TRPC5 | 2 |
| MF | GO:0008094 | ATP-dependent activity, acting on DNA | 4/98 | 111/18368 | 0.002951 | 0.110299 | 0.100426 | HFM1/DNA2/CDK7/SMARCA1 | 4 |
| MF | GO:0016888 | endodeoxyribonuclease activity, producing 5'-phosphomonoesters | 2/98 | 16/18368 | 0.00322 | 0.110299 | 0.100426 | DNA2/EXO1 | 2 |
| MF | GO:0008569 | minus-end-directed microtubule motor activity | 2/98 | 18/18368 | 0.004078 | 0.12414 | 0.113028 | DNAH11/DNAH6 | 2 |
| MF | GO:0016638 | oxidoreductase activity, acting on the CH-NH2 group of donors | 2/98 | 20/18368 | 0.005029 | 0.137279 | 0.124991 | GLUD1/RNLS | 2 |
| MF | GO:0097110 | scaffold protein binding | 3/98 | 67/18368 | 0.005511 | 0.137279 | 0.124991 | CIT/ITPR2/BRAF | 3 |
| MF | GO:0140097 | catalytic activity, acting on DNA | 5/98 | 229/18368 | 0.007663 | 0.174969 | 0.159308 | HFM1/DNA2/EXO1/CDK7/SMARCA1 | 5 |
| MF | GO:0051959 | dynein light intermediate chain binding | 2/98 | 27/18368 | 0.009067 | 0.181146 | 0.164931 | DNAH11/DNAH6 | 2 |
| MF | GO:0050681 | androgen receptor binding | 2/98 | 28/18368 | 0.009731 | 0.181146 | 0.164931 | FOXP2/PRKCB | 2 |
| MF | GO:0042393 | histone binding | 5/98 | 244/18368 | 0.009917 | 0.181146 | 0.164931 | TBL1XR1/USP49/KMT2C/PRKCB/TBL1X | 5 |
| MF | GO:0008013 | beta-catenin binding | 3/98 | 85/18368 | 0.010607 | 0.181649 | 0.16539 | TBL1XR1/CTNND2/TBL1X | 3 |
| MF | GO:0043178 | alcohol binding | 3/98 | 89/18368 | 0.012011 | 0.193596 | 0.176267 | TPK1/ITPR2/TRPC5 | 3 |
| MF | GO:0030291 | protein serine/threonine kinase inhibitor activity | 2/98 | 34/18368 | 0.014147 | 0.204007 | 0.185746 | RPTOR/CIT | 2 |
| MF | GO:0045505 | dynein intermediate chain binding | 2/98 | 34/18368 | 0.014147 | 0.204007 | 0.185746 | DNAH11/DNAH6 | 2 |
| MF | GO:0004520 | endodeoxyribonuclease activity | 2/98 | 38/18368 | 0.017485 | 0.233628 | 0.212715 | DNA2/EXO1 | 2 |
| MF | GO:0003714 | transcription corepressor activity | 4/98 | 187/18368 | 0.017906 | 0.233628 | 0.212715 | WWTR1/TBL1XR1/ZFPM2/TBL1X | 4 |
| MF | GO:0042626 | ATPase-coupled transmembrane transporter activity | 3/98 | 109/18368 | 0.020579 | 0.251073 | 0.228599 | ABCB7/ABCB5/ATP7A | 3 |
| MF | GO:0047485 | protein N-terminus binding | 3/98 | 110/18368 | 0.021075 | 0.251073 | 0.228599 | TBL1XR1/CSNK2A1/EXOC4 | 3 |
| MF | GO:0018024 | histone-lysine N-methyltransferase activity | 2/98 | 43/18368 | 0.022076 | 0.252039 | 0.229478 | MECOM/KMT2C | 2 |
| MF | GO:0016893 | endonuclease activity, active with either ribo- or deoxyribonucleic acids and producing 5'-phosphomonoesters | 2/98 | 45/18368 | 0.024038 | 0.263454 | 0.239872 | DNA2/EXO1 | 2 |
| MF | GO:0005262 | calcium channel activity | 3/98 | 119/18368 | 0.025843 | 0.272347 | 0.247969 | ITPR2/TRPC4AP/TRPC5 | 3 |
| MF | GO:0140359 | ABC-type transporter activity | 2/98 | 50/18368 | 0.029238 | 0.296715 | 0.270156 | ABCB7/ABCB5 | 2 |
| BP | GO:0018107 | peptidyl-threonine phosphorylation | 6/98 | 116/18723 | 3.29E-05 | 0.025236 | 0.02509 | NLK/RPTOR/CIT/PRKCB/CSNK2A1/TRPC5 | 6 |
| BP | GO:0018105 | peptidyl-serine phosphorylation | 9/98 | 315/18723 | 4.08E-05 | 0.025236 | 0.02509 | NLK/RPTOR/SPTBN4/MAST4/MARK3/PRKCB/AKT2/CSNK2A1/BRAF | 9 |
| BP | GO:0018210 | peptidyl-threonine modification | 6/98 | 125/18723 | 5.00E-05 | 0.025236 | 0.02509 | NLK/RPTOR/CIT/PRKCB/CSNK2A1/TRPC5 | 6 |
| BP | GO:0018209 | peptidyl-serine modification | 9/98 | 338/18723 | 7.03E-05 | 0.026593 | 0.026438 | NLK/RPTOR/SPTBN4/MAST4/MARK3/PRKCB/AKT2/CSNK2A1/BRAF | 9 |
| BP | GO:0035329 | hippo signaling | 3/98 | 40/18723 | 0.001194 | 0.274738 | 0.273142 | WWTR1/CIT/MARK3 | 3 |
| BP | GO:0015886 | heme transport | 2/98 | 11/18723 | 0.001446 | 0.274738 | 0.273142 | SLC48A1/ABCB7 | 2 |
| BP | GO:0010459 | negative regulation of heart rate | 2/98 | 12/18723 | 0.00173 | 0.274738 | 0.273142 | SPTBN4/RNLS | 2 |
| BP | GO:0035331 | negative regulation of hippo signaling | 2/98 | 13/18723 | 0.002037 | 0.274738 | 0.273142 | CIT/MARK3 | 2 |
| BP | GO:0048820 | hair follicle maturation | 2/98 | 13/18723 | 0.002037 | 0.274738 | 0.273142 | SPINK5/TRPC4AP | 2 |
| BP | GO:0000041 | transition metal ion transport | 4/98 | 105/18723 | 0.002252 | 0.274738 | 0.273142 | SLC48A1/ABCB7/ATP7A/TRPC5 | 4 |
| BP | GO:1901678 | iron coordination entity transport | 2/98 | 14/18723 | 0.002369 | 0.274738 | 0.273142 | SLC48A1/ABCB7 | 2 |
| BP | GO:0045927 | positive regulation of growth | 6/98 | 259/18723 | 0.002409 | 0.274738 | 0.273142 | RPTOR/SPTBN4/ZFPM2/RIMS2/CSNK2A1/TRPC5 | 6 |
| BP | GO:0001325 | formation of extrachromosomal circular DNA | 2/98 | 15/18723 | 0.002724 | 0.274738 | 0.273142 | DNA2/EXO1 | 2 |
| BP | GO:0090656 | t-circle formation | 2/98 | 15/18723 | 0.002724 | 0.274738 | 0.273142 | DNA2/EXO1 | 2 |
| BP | GO:0090737 | telomere maintenance via telomere trimming | 2/98 | 15/18723 | 0.002724 | 0.274738 | 0.273142 | DNA2/EXO1 | 2 |
| CC | GO:0005938 | cell cortex | 7/99 | 308/19550 | 0.000972 | 0.210043 | 0.195508 | SPTBN4/SPINK5/RIMS2/ITPR2/PRKCB/AKT2/EXOC4 | 7 |
| CC | GO:0000159 | protein phosphatase type 2A complex | 2/99 | 17/19550 | 0.003285 | 0.280653 | 0.261232 | PPP2R3A/PPP2R2B | 2 |
| CC | GO:0000118 | histone deacetylase complex | 3/99 | 72/19550 | 0.005835 | 0.280653 | 0.261232 | TBL1XR1/CSNK2A1/TBL1X | 3 |
| CC | GO:0048786 | presynaptic active zone | 3/99 | 73/19550 | 0.006063 | 0.280653 | 0.261232 | SYN1/RIMS2/PPFIBP1 | 3 |
| CC | GO:0005963 | magnesium-dependent protein serine/threonine phosphatase complex | 2/99 | 26/19550 | 0.007622 | 0.280653 | 0.261232 | PPP2R3A/PPP2R2B | 2 |
| CC | GO:0005802 | trans-Golgi network | 5/99 | 259/19550 | 0.010236 | 0.280653 | 0.261232 | CCDC91/SLC10A7/COG5/CLVS1/ATP7A | 5 |
| CC | GO:0016342 | catenin complex | 2/99 | 31/19550 | 0.010728 | 0.280653 | 0.261232 | CDH12/CDH18 | 2 |
| CC | GO:0005912 | adherens junction | 4/99 | 170/19550 | 0.010958 | 0.280653 | 0.261232 | SPTBN4/CDH12/CTNND2/CDH18 | 4 |
| CC | GO:0080008 | Cul4-RING E3 ubiquitin ligase complex | 2/99 | 34/19550 | 0.012817 | 0.280653 | 0.261232 | DCAF8/TRPC4AP | 2 |
| CC | GO:0098791 | Golgi apparatus subcompartment | 6/99 | 384/19550 | 0.01341 | 0.280653 | 0.261232 | CCDC91/B4GALT6/SLC10A7/COG5/CLVS1/ATP7A | 6 |
| CC | GO:0032588 | trans-Golgi network membrane | 3/99 | 100/19550 | 0.014293 | 0.280653 | 0.261232 | COG5/CLVS1/ATP7A | 3 |
| CC | GO:0033017 | sarcoplasmic reticulum membrane | 2/99 | 40/19550 | 0.017474 | 0.286007 | 0.266215 | NOS1AP/ITPR2 | 2 |
| CC | GO:0030863 | cortical cytoskeleton | 3/99 | 108/19550 | 0.017532 | 0.286007 | 0.266215 | SPTBN4/RIMS2/PRKCB | 3 |
| CC | GO:0005667 | transcription regulator complex | 6/99 | 413/19550 | 0.018537 | 0.286007 | 0.266215 | WWTR1/TBL1XR1/CSNK2A1/CDK7/TBL1X/DACH2 | 6 |
| KEGG | hsa04310 | Wnt signaling pathway | 7/41 | 167/8113 | 1.72E-05 | 0.00315 | 0.002464 | NLK/TBL1XR1/WNT16/CTNND2/PRKCB/CSNK2A1/TBL1X | 7 |
| KEGG | hsa04929 | GnRH secretion | 4/41 | 64/8113 | 0.000287 | 0.026218 | 0.02051 | ITPR2/PRKCB/AKT2/TRPC5 | 4 |
| KEGG | hsa04728 | Dopaminergic synapse | 5/41 | 132/8113 | 0.000495 | 0.030191 | 0.023618 | PPP2R3A/PPP2R2B/ITPR2/PRKCB/AKT2 | 5 |
| KEGG | hsa04150 | mTOR signaling pathway | 5/41 | 155/8113 | 0.001026 | 0.04696 | 0.036736 | RPTOR/WNT16/PRKCB/AKT2/BRAF | 5 |
| KEGG | hsa05022 | Pathways of neurodegeneration - multiple diseases | 8/41 | 476/8113 | 0.002271 | 0.07133 | 0.0558 | PSMA1/DNAH11/WNT16/DNAH6/ITPR2/PRKCB/CSNK2A1/BRAF | 8 |
| KEGG | hsa04071 | Sphingolipid signaling pathway | 4/41 | 119/8113 | 0.002932 | 0.07133 | 0.0558 | PPP2R3A/PPP2R2B/PRKCB/AKT2 | 4 |
| KEGG | hsa04152 | AMPK signaling pathway | 4/41 | 120/8113 | 0.003022 | 0.07133 | 0.0558 | RPTOR/PPP2R3A/PPP2R2B/AKT2 | 4 |
| KEGG | hsa04730 | Long-term depression | 3/41 | 60/8113 | 0.003357 | 0.07133 | 0.0558 | ITPR2/PRKCB/BRAF | 3 |
| KEGG | hsa05205 | Proteoglycans in cancer | 5/41 | 205/8113 | 0.003508 | 0.07133 | 0.0558 | WNT16/ITPR2/PRKCB/AKT2/BRAF | 5 |
| KEGG | hsa04720 | Long-term potentiation | 3/41 | 67/8113 | 0.004587 | 0.074607 | 0.058364 | ITPR2/PRKCB/BRAF | 3 |
| KEGG | hsa04520 | Adherens junction | 3/41 | 71/8113 | 0.005397 | 0.074607 | 0.058364 | NLK/LMO7/CSNK2A1 | 3 |
| KEGG | hsa05223 | Non-small cell lung cancer | 3/41 | 72/8113 | 0.005612 | 0.074607 | 0.058364 | PRKCB/AKT2/BRAF | 3 |
| KEGG | hsa05017 | Spinocerebellar ataxia | 4/41 | 143/8113 | 0.005652 | 0.074607 | 0.058364 | PSMA1/ITPR2/PRKCB/AKT2 | 4 |
| KEGG | hsa01524 | Platinum drug resistance | 3/41 | 73/8113 | 0.005832 | 0.074607 | 0.058364 | BIRC3/AKT2/ATP7A | 3 |
| KEGG | hsa05214 | Glioma | 3/41 | 75/8113 | 0.006287 | 0.074607 | 0.058364 | PRKCB/AKT2/BRAF | 3 |
| KEGG | hsa05220 | Chronic myeloid leukemia | 3/41 | 76/8113 | 0.006523 | 0.074607 | 0.058364 | MECOM/AKT2/BRAF | 3 |
| KEGG | hsa01521 | EGFR tyrosine kinase inhibitor resistance | 3/41 | 79/8113 | 0.007261 | 0.078166 | 0.061148 | PRKCB/AKT2/BRAF | 3 |
| KEGG | hsa04390 | Hippo signaling pathway | 4/41 | 157/8113 | 0.007835 | 0.079659 | 0.062316 | BIRC3/WWTR1/WNT16/PPP2R2B | 4 |
| KEGG | hsa04012 | ErbB signaling pathway | 3/41 | 85/8113 | 0.008882 | 0.085548 | 0.066923 | PRKCB/AKT2/BRAF | 3 |
| KEGG | hsa05225 | Hepatocellular carcinoma | 4/41 | 168/8113 | 0.009897 | 0.090558 | 0.070842 | WNT16/PRKCB/AKT2/BRAF | 4 |
| KEGG | hsa05010 | Alzheimer disease | 6/41 | 384/8113 | 0.01193 | 0.103958 | 0.081325 | PSMA1/WNT16/ITPR2/AKT2/CSNK2A1/BRAF | 6 |
| KEGG | hsa04064 | NF-kappa B signaling pathway | 3/41 | 104/8113 | 0.015331 | 0.122982 | 0.096207 | BIRC3/PRKCB/CSNK2A1 | 3 |
| KEGG | hsa04010 | MAPK signaling pathway | 5/41 | 294/8113 | 0.015553 | 0.122982 | 0.096207 | NLK/MECOM/PRKCB/AKT2/BRAF | 5 |
| KEGG | hsa04928 | Parathyroid hormone synthesis, secretion and action | 3/41 | 106/8113 | 0.016129 | 0.122982 | 0.096207 | ITPR2/PRKCB/BRAF | 3 |
| KEGG | hsa04510 | Focal adhesion | 4/41 | 201/8113 | 0.018107 | 0.132547 | 0.103689 | BIRC3/PRKCB/AKT2/BRAF | 4 |
| KEGG | hsa04725 | Cholinergic synapse | 3/41 | 113/8113 | 0.019106 | 0.134475 | 0.105198 | ITPR2/PRKCB/AKT2 | 3 |
| KEGG | hsa04726 | Serotonergic synapse | 3/41 | 115/8113 | 0.020009 | 0.135615 | 0.106089 | ITPR2/PRKCB/BRAF | 3 |
| KEGG | hsa02010 | ABC transporters | 2/41 | 45/8113 | 0.021508 | 0.138101 | 0.108034 | ABCB7/ABCB5 | 2 |
| KEGG | hsa04935 | Growth hormone synthesis, secretion and action | 3/41 | 119/8113 | 0.021885 | 0.138101 | 0.108034 | ITPR2/PRKCB/AKT2 | 3 |
| KEGG | hsa04973 | Carbohydrate digestion and absorption | 2/41 | 47/8113 | 0.023337 | 0.142357 | 0.111364 | PRKCB/AKT2 | 2 |
| KEGG | hsa04068 | FoxO signaling pathway | 3/41 | 131/8113 | 0.028076 | 0.165737 | 0.129654 | NLK/AKT2/BRAF | 3 |
| KEGG | hsa04270 | Vascular smooth muscle contraction | 3/41 | 134/8113 | 0.029755 | 0.169475 | 0.132577 | ITPR2/PRKCB/BRAF | 3 |
| KEGG | hsa04210 | Apoptosis | 3/41 | 136/8113 | 0.030904 | 0.169475 | 0.132577 | BIRC3/ITPR2/AKT2 | 3 |
| KEGG | hsa04910 | Insulin signaling pathway | 3/41 | 137/8113 | 0.031487 | 0.169475 | 0.132577 | RPTOR/AKT2/BRAF | 3 |
| KEGG | hsa05213 | Endometrial cancer | 2/41 | 58/8113 | 0.034467 | 0.17466 | 0.136634 | AKT2/BRAF | 2 |
| KEGG | hsa04370 | VEGF signaling pathway | 2/41 | 59/8113 | 0.035564 | 0.17466 | 0.136634 | PRKCB/AKT2 | 2 |
| KEGG | hsa05132 | Salmonella infection | 4/41 | 249/8113 | 0.036088 | 0.17466 | 0.136634 | BIRC3/AKT2/ARHGEF26/EXOC4 | 4 |
| KEGG | hsa05224 | Breast cancer | 3/41 | 147/8113 | 0.037638 | 0.17466 | 0.136634 | WNT16/AKT2/BRAF | 3 |
| KEGG | hsa04213 | Longevity regulating pathway - multiple species | 2/41 | 62/8113 | 0.038936 | 0.17466 | 0.136634 | RPTOR/AKT2 | 2 |
| KEGG | hsa05226 | Gastric cancer | 3/41 | 149/8113 | 0.038937 | 0.17466 | 0.136634 | WNT16/AKT2/BRAF | 3 |
| KEGG | hsa04261 | Adrenergic signaling in cardiomyocytes | 3/41 | 150/8113 | 0.039595 | 0.17466 | 0.136634 | PPP2R3A/PPP2R2B/AKT2 | 3 |
| KEGG | hsa00310 | Lysine degradation | 2/41 | 63/8113 | 0.040086 | 0.17466 | 0.136634 | MECOM/KMT2C | 2 |
| KEGG | hsa04934 | Cushing syndrome | 3/41 | 155/8113 | 0.042972 | 0.182244 | 0.142566 | WNT16/ITPR2/BRAF | 3 |
| KEGG | hsa05160 | Hepatitis C | 3/41 | 157/8113 | 0.044362 | 0.182244 | 0.142566 | PPP2R2B/AKT2/BRAF | 3 |
| KEGG | hsa05221 | Acute myeloid leukemia | 2/41 | 67/8113 | 0.044814 | 0.182244 | 0.142566 | AKT2/BRAF | 2 |
| KEGG | hsa05211 | Renal cell carcinoma | 2/41 | 69/8113 | 0.047252 | 0.186643 | 0.146008 | AKT2/BRAF | 2 |
| KEGG | hsa05161 | Hepatitis B | 3/41 | 162/8113 | 0.047936 | 0.186643 | 0.146008 | PRKCB/AKT2/BRAF | 3 |
| KEGG | hsa05218 | Melanoma | 2/41 | 72/8113 | 0.050999 | 0.194435 | 0.152103 | AKT2/BRAF | 2 |
| KEGG | hsa04918 | Thyroid hormone synthesis | 2/41 | 75/8113 | 0.05485 | 0.201503 | 0.157633 | ITPR2/PRKCB | 2 |
| KEGG | hsa04971 | Gastric acid secretion | 2/41 | 76/8113 | 0.056157 | 0.201503 | 0.157633 | ITPR2/PRKCB | 2 |
| KEGG | hsa05212 | Pancreatic cancer | 2/41 | 76/8113 | 0.056157 | 0.201503 | 0.157633 | AKT2/BRAF | 2 |
| KEGG | hsa04662 | B cell receptor signaling pathway | 2/41 | 82/8113 | 0.06422 | 0.214084 | 0.167474 | PRKCB/AKT2 | 2 |
| KEGG | hsa04911 | Insulin secretion | 2/41 | 86/8113 | 0.0698 | 0.214084 | 0.167474 | RIMS2/PRKCB | 2 |
| KEGG | hsa05210 | Colorectal cancer | 2/41 | 86/8113 | 0.0698 | 0.214084 | 0.167474 | AKT2/BRAF | 2 |
| KEGG | hsa04062 | Chemokine signaling pathway | 3/41 | 192/8113 | 0.072229 | 0.214084 | 0.167474 | PRKCB/AKT2/BRAF | 3 |
| KEGG | hsa04540 | Gap junction | 2/41 | 88/8113 | 0.072649 | 0.214084 | 0.167474 | ITPR2/PRKCB | 2 |
| KEGG | hsa00730 | Thiamine metabolism | 1/41 | 15/8113 | 0.073242 | 0.214084 | 0.167474 | TPK1 | 1 |
| KEGG | hsa04211 | Longevity regulating pathway | 2/41 | 89/8113 | 0.074088 | 0.214084 | 0.167474 | RPTOR/AKT2 | 2 |
| KEGG | hsa05235 | PD-L1 expression and PD-1 checkpoint pathway in cancer | 2/41 | 89/8113 | 0.074088 | 0.214084 | 0.167474 | AKT2/CSNK2A1 | 2 |
| KEGG | hsa05222 | Small cell lung cancer | 2/41 | 92/8113 | 0.078458 | 0.214084 | 0.167474 | BIRC3/AKT2 | 2 |
| KEGG | hsa04912 | GnRH signaling pathway | 2/41 | 93/8113 | 0.079933 | 0.214084 | 0.167474 | ITPR2/PRKCB | 2 |
| KEGG | hsa04970 | Salivary secretion | 2/41 | 93/8113 | 0.079933 | 0.214084 | 0.167474 | ITPR2/PRKCB | 2 |
| KEGG | hsa00910 | Nitrogen metabolism | 1/41 | 17/8113 | 0.082603 | 0.214084 | 0.167474 | GLUD1 | 1 |
| KEGG | hsa05165 | Human papillomavirus infection | 4/41 | 331/8113 | 0.084563 | 0.214084 | 0.167474 | WNT16/PPP2R3A/PPP2R2B/AKT2 | 4 |
| KEGG | hsa03015 | mRNA surveillance pathway | 2/41 | 97/8113 | 0.08592 | 0.214084 | 0.167474 | PPP2R3A/PPP2R2B | 2 |
| KEGG | hsa04070 | Phosphatidylinositol signaling system | 2/41 | 97/8113 | 0.08592 | 0.214084 | 0.167474 | ITPR2/PRKCB | 2 |
| KEGG | hsa04666 | Fc gamma R-mediated phagocytosis | 2/41 | 97/8113 | 0.08592 | 0.214084 | 0.167474 | PRKCB/AKT2 | 2 |
| KEGG | hsa04713 | Circadian entrainment | 2/41 | 97/8113 | 0.08592 | 0.214084 | 0.167474 | NOS1AP/PRKCB | 2 |
| KEGG | hsa05215 | Prostate cancer | 2/41 | 97/8113 | 0.08592 | 0.214084 | 0.167474 | AKT2/BRAF | 2 |
| KEGG | hsa01522 | Endocrine resistance | 2/41 | 98/8113 | 0.087437 | 0.214084 | 0.167474 | AKT2/BRAF | 2 |
| KEGG | hsa04750 | Inflammatory mediator regulation of TRP channels | 2/41 | 98/8113 | 0.087437 | 0.214084 | 0.167474 | ITPR2/PRKCB | 2 |
| KEGG | hsa04925 | Aldosterone synthesis and secretion | 2/41 | 98/8113 | 0.087437 | 0.214084 | 0.167474 | ITPR2/PRKCB | 2 |
| KEGG | hsa05231 | Choline metabolism in cancer | 2/41 | 98/8113 | 0.087437 | 0.214084 | 0.167474 | PRKCB/AKT2 | 2 |
| KEGG | hsa04015 | Rap1 signaling pathway | 3/41 | 210/8113 | 0.089008 | 0.214084 | 0.167474 | PRKCB/AKT2/BRAF | 3 |
| KEGG | hsa04933 | AGE-RAGE signaling pathway in diabetic complications | 2/41 | 100/8113 | 0.090497 | 0.214084 | 0.167474 | PRKCB/AKT2 | 2 |
| KEGG | hsa05170 | Human immunodeficiency virus 1 infection | 3/41 | 212/8113 | 0.090968 | 0.214084 | 0.167474 | ITPR2/PRKCB/AKT2 | 3 |
| KEGG | hsa04916 | Melanogenesis | 2/41 | 101/8113 | 0.092039 | 0.214084 | 0.167474 | WNT16/PRKCB | 2 |
| KEGG | hsa04914 | Progesterone-mediated oocyte maturation | 2/41 | 102/8113 | 0.093589 | 0.214084 | 0.167474 | AKT2/BRAF | 2 |
| KEGG | hsa04972 | Pancreatic secretion | 2/41 | 102/8113 | 0.093589 | 0.214084 | 0.167474 | ITPR2/PRKCB | 2 |
| KEGG | hsa05142 | Chagas disease | 2/41 | 102/8113 | 0.093589 | 0.214084 | 0.167474 | PPP2R2B/AKT2 | 2 |
| KEGG | hsa04625 | C-type lectin receptor signaling pathway | 2/41 | 104/8113 | 0.096711 | 0.218495 | 0.170925 | ITPR2/AKT2 | 2 |
| KEGG | hsa04922 | Glucagon signaling pathway | 2/41 | 107/8113 | 0.10145 | 0.221482 | 0.173262 | ITPR2/AKT2 | 2 |
| KEGG | hsa04151 | PI3K-Akt signaling pathway | 4/41 | 354/8113 | 0.102071 | 0.221482 | 0.173262 | RPTOR/PPP2R3A/PPP2R2B/AKT2 | 4 |
| KEGG | hsa04931 | Insulin resistance | 2/41 | 108/8113 | 0.103045 | 0.221482 | 0.173262 | PRKCB/AKT2 | 2 |
| KEGG | hsa05163 | Human cytomegalovirus infection | 3/41 | 225/8113 | 0.104143 | 0.221482 | 0.173262 | ITPR2/PRKCB/AKT2 | 3 |
| KEGG | hsa04066 | HIF-1 signaling pathway | 2/41 | 109/8113 | 0.104646 | 0.221482 | 0.173262 | PRKCB/AKT2 | 2 |
| KEGG | hsa00220 | Arginine biosynthesis | 1/41 | 22/8113 | 0.105604 | 0.221482 | 0.173262 | GLUD1 | 1 |
| KEGG | hsa04668 | TNF signaling pathway | 2/41 | 112/8113 | 0.109492 | 0.221482 | 0.173262 | BIRC3/AKT2 | 2 |
| KEGG | hsa05145 | Toxoplasmosis | 2/41 | 112/8113 | 0.109492 | 0.221482 | 0.173262 | BIRC3/AKT2 | 2 |
| KEGG | hsa03430 | Mismatch repair | 1/41 | 23/8113 | 0.110136 | 0.221482 | 0.173262 | EXO1 | 1 |
| KEGG | hsa04964 | Proximal tubule bicarbonate reclamation | 1/41 | 23/8113 | 0.110136 | 0.221482 | 0.173262 | GLUD1 | 1 |
| KEGG | hsa04724 | Glutamatergic synapse | 2/41 | 114/8113 | 0.112755 | 0.224285 | 0.175455 | ITPR2/PRKCB | 2 |
| KEGG | hsa04722 | Neurotrophin signaling pathway | 2/41 | 119/8113 | 0.121025 | 0.238147 | 0.186298 | AKT2/BRAF | 2 |
| KEGG | hsa04919 | Thyroid hormone signaling pathway | 2/41 | 121/8113 | 0.124375 | 0.242135 | 0.189418 | PRKCB/AKT2 | 2 |
| KEGG | hsa05131 | Shigellosis | 3/41 | 247/8113 | 0.128054 | 0.246672 | 0.192967 | RPTOR/ITPR2/AKT2 | 3 |
| KEGG | hsa04611 | Platelet activation | 2/41 | 124/8113 | 0.129443 | 0.246751 | 0.19303 | ITPR2/AKT2 | 2 |
| KEGG | hsa04392 | Hippo signaling pathway - multiple species | 1/41 | 29/8113 | 0.136862 | 0.258204 | 0.201989 | WWTR1 | 1 |
| KEGG | hsa04650 | Natural killer cell mediated cytotoxicity | 2/41 | 131/8113 | 0.141454 | 0.264144 | 0.206636 | PRKCB/BRAF | 2 |
| KEGG | hsa04136 | Autophagy - other | 1/41 | 32/8113 | 0.14993 | 0.274373 | 0.214637 | RPTOR | 1 |
| KEGG | hsa04215 | Apoptosis - multiple species | 1/41 | 32/8113 | 0.14993 | 0.274373 | 0.214637 | BIRC3 | 1 |
| KEGG | hsa04915 | Estrogen signaling pathway | 2/41 | 138/8113 | 0.1537 | 0.276219 | 0.216081 | ITPR2/AKT2 | 2 |
| KEGG | hsa04371 | Apelin signaling pathway | 2/41 | 139/8113 | 0.155467 | 0.276219 | 0.216081 | ITPR2/AKT2 | 2 |
| KEGG | hsa05162 | Measles | 2/41 | 139/8113 | 0.155467 | 0.276219 | 0.216081 | AKT2/CSNK2A1 | 2 |
| KEGG | hsa05020 | Prion disease | 3/41 | 273/8113 | 0.158608 | 0.277137 | 0.2168 | PSMA1/ITPR2/CSNK2A1 | 3 |
| KEGG | hsa04140 | Autophagy - animal | 2/41 | 141/8113 | 0.159013 | 0.277137 | 0.2168 | RPTOR/AKT2 | 2 |
| KEGG | hsa04120 | Ubiquitin mediated proteolysis | 2/41 | 142/8113 | 0.160792 | 0.277594 | 0.217157 | BIRC3/MID1 | 2 |
| KEGG | hsa04550 | Signaling pathways regulating pluripotency of stem cells | 2/41 | 143/8113 | 0.162575 | 0.27781 | 0.217326 | WNT16/AKT2 | 2 |
| KEGG | hsa03030 | DNA replication | 1/41 | 36/8113 | 0.167055 | 0.27781 | 0.217326 | DNA2 | 1 |
| KEGG | hsa00250 | Alanine, aspartate and glutamate metabolism | 1/41 | 37/8113 | 0.171283 | 0.27781 | 0.217326 | GLUD1 | 1 |
| KEGG | hsa04960 | Aldosterone-regulated sodium reabsorption | 1/41 | 37/8113 | 0.171283 | 0.27781 | 0.217326 | PRKCB | 1 |
| KEGG | hsa05143 | African trypanosomiasis | 1/41 | 37/8113 | 0.171283 | 0.27781 | 0.217326 | PRKCB | 1 |
| KEGG | hsa05216 | Thyroid cancer | 1/41 | 37/8113 | 0.171283 | 0.27781 | 0.217326 | BRAF | 1 |
| KEGG | hsa04723 | Retrograde endocannabinoid signaling | 2/41 | 148/8113 | 0.171544 | 0.27781 | 0.217326 | ITPR2/PRKCB | 2 |
| KEGG | hsa04921 | Oxytocin signaling pathway | 2/41 | 154/8113 | 0.182417 | 0.292827 | 0.229073 | ITPR2/PRKCB | 2 |
| KEGG | hsa04218 | Cellular senescence | 2/41 | 156/8113 | 0.186065 | 0.296086 | 0.231623 | ITPR2/AKT2 | 2 |
| KEGG | hsa05219 | Bladder cancer | 1/41 | 41/8113 | 0.187987 | 0.296565 | 0.231998 | BRAF | 1 |
| KEGG | hsa04217 | Necroptosis | 2/41 | 159/8113 | 0.191558 | 0.299617 | 0.234385 | GLUD1/BIRC3 | 2 |
| KEGG | hsa05016 | Huntington disease | 3/41 | 306/8113 | 0.200285 | 0.310612 | 0.242987 | PSMA1/DNAH11/DNAH6 | 3 |
| KEGG | hsa03022 | Basal transcription factors | 1/41 | 45/8113 | 0.204362 | 0.31203 | 0.244096 | CDK7 | 1 |
| KEGG | hsa05206 | MicroRNAs in cancer | 3/41 | 310/8113 | 0.205517 | 0.31203 | 0.244096 | RPTOR/ZFPM2/PRKCB | 3 |
| KEGG | hsa04022 | cGMP-PKG signaling pathway | 2/41 | 167/8113 | 0.206315 | 0.31203 | 0.244096 | ITPR2/AKT2 | 2 |
| KEGG | hsa03050 | Proteasome | 1/41 | 46/8113 | 0.208406 | 0.312608 | 0.244548 | PSMA1 | 1 |
| KEGG | hsa03420 | Nucleotide excision repair | 1/41 | 47/8113 | 0.212429 | 0.315444 | 0.246766 | CDK7 | 1 |
| KEGG | hsa05164 | Influenza A | 2/41 | 171/8113 | 0.213743 | 0.315444 | 0.246766 | PRKCB/AKT2 | 2 |
| KEGG | hsa00600 | Sphingolipid metabolism | 1/41 | 49/8113 | 0.220416 | 0.322688 | 0.252434 | B4GALT6 | 1 |
| KEGG | hsa04961 | Endocrine and other factor-regulated calcium reabsorption | 1/41 | 53/8113 | 0.236153 | 0.342984 | 0.26831 | PRKCB | 1 |
| KEGG | hsa04621 | NOD-like receptor signaling pathway | 2/41 | 184/8113 | 0.23806 | 0.343031 | 0.268347 | BIRC3/ITPR2 | 2 |
| KEGG | hsa04923 | Regulation of lipolysis in adipocytes | 1/41 | 56/8113 | 0.247752 | 0.353723 | 0.276712 | AKT2 | 1 |
| KEGG | hsa04613 | Neutrophil extracellular trap formation | 2/41 | 190/8113 | 0.249346 | 0.353723 | 0.276712 | PRKCB/AKT2 | 2 |
| KEGG | hsa05202 | Transcriptional misregulation in cancer | 2/41 | 192/8113 | 0.253114 | 0.356306 | 0.278733 | BIRC3/WNT16 | 2 |
| KEGG | hsa05167 | Kaposi sarcoma-associated herpesvirus infection | 2/41 | 194/8113 | 0.256884 | 0.358853 | 0.280725 | ITPR2/AKT2 | 2 |
| KEGG | hsa04978 | Mineral absorption | 1/41 | 60/8113 | 0.26295 | 0.364544 | 0.285177 | ATP7A | 1 |
| KEGG | hsa05415 | Diabetic cardiomyopathy | 2/41 | 203/8113 | 0.273865 | 0.374401 | 0.292888 | PRKCB/AKT2 | 2 |
| KEGG | hsa05217 | Basal cell carcinoma | 1/41 | 63/8113 | 0.274152 | 0.374401 | 0.292888 | WNT16 | 1 |
| KEGG | hsa05014 | Amyotrophic lateral sclerosis | 3/41 | 364/8113 | 0.278786 | 0.37791 | 0.295632 | PSMA1/DNAH11/DNAH6 | 3 |
| KEGG | hsa04927 | Cortisol synthesis and secretion | 1/41 | 65/8113 | 0.281527 | 0.378819 | 0.296344 | ITPR2 | 1 |
| KEGG | hsa00970 | Aminoacyl-tRNA biosynthesis | 1/41 | 66/8113 | 0.285187 | 0.380801 | 0.297895 | FARS2 | 1 |
| KEGG | hsa05207 | Chemical carcinogenesis - receptor activation | 2/41 | 212/8113 | 0.290845 | 0.380801 | 0.297895 | PRKCB/AKT2 | 2 |
| KEGG | hsa04664 | Fc epsilon RI signaling pathway | 1/41 | 68/8113 | 0.292453 | 0.380801 | 0.297895 | AKT2 | 1 |
| KEGG | hsa04920 | Adipocytokine signaling pathway | 1/41 | 69/8113 | 0.296059 | 0.380801 | 0.297895 | AKT2 | 1 |
| KEGG | hsa04924 | Renin secretion | 1/41 | 69/8113 | 0.296059 | 0.380801 | 0.297895 | ITPR2 | 1 |
| KEGG | hsa05031 | Amphetamine addiction | 1/41 | 69/8113 | 0.296059 | 0.380801 | 0.297895 | PRKCB | 1 |
| KEGG | hsa04917 | Prolactin signaling pathway | 1/41 | 70/8113 | 0.299647 | 0.380801 | 0.297895 | AKT2 | 1 |
| KEGG | hsa05230 | Central carbon metabolism in cancer | 1/41 | 70/8113 | 0.299647 | 0.380801 | 0.297895 | AKT2 | 1 |
| KEGG | hsa04810 | Regulation of actin cytoskeleton | 2/41 | 218/8113 | 0.302149 | 0.381332 | 0.29831 | DIAPH2/BRAF | 2 |
| KEGG | hsa04137 | Mitophagy - animal | 1/41 | 72/8113 | 0.306769 | 0.38317 | 0.299747 | CSNK2A1 | 1 |
| KEGG | hsa04024 | cAMP signaling pathway | 2/41 | 221/8113 | 0.307792 | 0.38317 | 0.299747 | AKT2/BRAF | 2 |
| KEGG | hsa05208 | Chemical carcinogenesis - reactive oxygen species | 2/41 | 223/8113 | 0.31155 | 0.385228 | 0.301357 | AKT2/BRAF | 2 |
| KEGG | hsa05100 | Bacterial invasion of epithelial cells | 1/41 | 77/8113 | 0.324268 | 0.395607 | 0.309477 | ARHGEF26 | 1 |
| KEGG | hsa05140 | Leishmaniasis | 1/41 | 77/8113 | 0.324268 | 0.395607 | 0.309477 | PRKCB | 1 |
| KEGG | hsa04014 | Ras signaling pathway | 2/41 | 232/8113 | 0.328416 | 0.398014 | 0.31136 | PRKCB/AKT2 | 2 |
| KEGG | hsa04020 | Calcium signaling pathway | 2/41 | 240/8113 | 0.343327 | 0.413347 | 0.323355 | ITPR2/PRKCB | 2 |
| KEGG | hsa04727 | GABAergic synapse | 1/41 | 89/8113 | 0.364525 | 0.436 | 0.341076 | PRKCB | 1 |
| KEGG | hsa05032 | Morphine addiction | 1/41 | 91/8113 | 0.371003 | 0.440867 | 0.344883 | PRKCB | 1 |
| KEGG | hsa05012 | Parkinson disease | 2/41 | 266/8113 | 0.391059 | 0.461702 | 0.361182 | PSMA1/ITPR2 | 2 |
| KEGG | hsa05146 | Amoebiasis | 1/41 | 102/8113 | 0.405496 | 0.475678 | 0.372115 | PRKCB | 1 |
| KEGG | hsa04620 | Toll-like receptor signaling pathway | 1/41 | 104/8113 | 0.411566 | 0.476687 | 0.372905 | AKT2 | 1 |
| KEGG | hsa04660 | T cell receptor signaling pathway | 1/41 | 104/8113 | 0.411566 | 0.476687 | 0.372905 | AKT2 | 1 |
| KEGG | hsa03008 | Ribosome biogenesis in eukaryotes | 1/41 | 109/8113 | 0.426478 | 0.490852 | 0.383986 | CSNK2A1 | 1 |
| KEGG | hsa04670 | Leukocyte transendothelial migration | 1/41 | 114/8113 | 0.441021 | 0.504418 | 0.394598 | PRKCB | 1 |
| KEGG | hsa01200 | Carbon metabolism | 1/41 | 115/8113 | 0.443886 | 0.504542 | 0.394695 | GLUD1 | 1 |
| KEGG | hsa05168 | Herpes simplex virus 1 infection | 3/41 | 495/8113 | 0.460913 | 0.520661 | 0.407304 | BIRC3/AKT2/ZNF182 | 3 |
| KEGG | hsa04110 | Cell cycle | 1/41 | 126/8113 | 0.474472 | 0.53269 | 0.416714 | CDK7 | 1 |
| KEGG | hsa04380 | Osteoclast differentiation | 1/41 | 128/8113 | 0.479854 | 0.535164 | 0.41865 | AKT2 | 1 |
| KEGG | hsa04926 | Relaxin signaling pathway | 1/41 | 129/8113 | 0.482525 | 0.535164 | 0.41865 | AKT2 | 1 |
| KEGG | hsa04114 | Oocyte meiosis | 1/41 | 131/8113 | 0.487826 | 0.537784 | 0.4207 | ITPR2 | 1 |
| KEGG | hsa05135 | Yersinia infection | 1/41 | 137/8113 | 0.503415 | 0.551646 | 0.431544 | AKT2 | 1 |
| KEGG | hsa05418 | Fluid shear stress and atherosclerosis | 1/41 | 139/8113 | 0.508507 | 0.55391 | 0.433314 | AKT2 | 1 |
| KEGG | hsa04936 | Alcoholic liver disease | 1/41 | 142/8113 | 0.51605 | 0.5588 | 0.43714 | AKT2 | 1 |
| KEGG | hsa04072 | Phospholipase D signaling pathway | 1/41 | 148/8113 | 0.5308 | 0.57139 | 0.446989 | AKT2 | 1 |
| KEGG | hsa01240 | Biosynthesis of cofactors | 1/41 | 155/8113 | 0.547454 | 0.582465 | 0.455653 | TPK1 | 1 |
| KEGG | hsa04932 | Non-alcoholic fatty liver disease | 1/41 | 155/8113 | 0.547454 | 0.582465 | 0.455653 | AKT2 | 1 |
| KEGG | hsa04630 | JAK-STAT signaling pathway | 1/41 | 162/8113 | 0.56353 | 0.596104 | 0.466323 | AKT2 | 1 |
| KEGG | hsa04530 | Tight junction | 1/41 | 169/8113 | 0.579049 | 0.609 | 0.476411 | PPP2R2B | 1 |
| KEGG | hsa05152 | Tuberculosis | 1/41 | 180/8113 | 0.602355 | 0.629891 | 0.492753 | AKT2 | 1 |
| KEGG | hsa04360 | Axon guidance | 1/41 | 182/8113 | 0.606455 | 0.630575 | 0.493289 | TRPC5 | 1 |
| KEGG | hsa05034 | Alcoholism | 1/41 | 187/8113 | 0.616525 | 0.637424 | 0.498646 | BRAF | 1 |
| KEGG | hsa05169 | Epstein-Barr virus infection | 1/41 | 202/8113 | 0.645251 | 0.663376 | 0.518948 | AKT2 | 1 |
| KEGG | hsa05417 | Lipid and atherosclerosis | 1/41 | 215/8113 | 0.668439 | 0.683377 | 0.534594 | AKT2 | 1 |
| KEGG | hsa05166 | Human T-cell leukemia virus 1 infection | 1/41 | 222/8113 | 0.680306 | 0.691645 | 0.541062 | AKT2 | 1 |
| KEGG | hsa04714 | Thermogenesis | 1/41 | 232/8113 | 0.696543 | 0.70037 | 0.547888 | RPTOR | 1 |
| KEGG | hsa05171 | Coronavirus disease - COVID-19 | 1/41 | 232/8113 | 0.696543 | 0.70037 | 0.547888 | PRKCB | 1 |
| KEGG | hsa04080 | Neuroactive ligand-receptor interaction | 1/41 | 353/8113 | 0.839348 | 0.839348 | 0.656608 | GPR156 | 1 |

BP, biological process; MF, molecular function; CC, cellular component. GO, Gene Ontology, KEGG, Kyoto Encyclopedia of Genes and Genomes.
